# Evolutionary Relationships of the *Phytophthora* 1a Subclade Species Based on Complete Mitogenomes, and Novel Markers for Their Differentiation

**DOI:** 10.1002/ece3.71105

**Published:** 2025-03-13

**Authors:** Tao Lin, Huiqin Wang, Haiting Ma, Jiaying Duan, Wenxin Wang, Xiaoyu Shi, Yaling Li, Zengqiang Qian, Nian Liu, Jiabin Zou, Ayaka Hieno, Koji Kageyama, Mingzhu Li

**Affiliations:** ^1^ The Key Laboratory of Medicinal Resources and Natural Pharmaceutical Chemistry, The Ministry of Education Shaanxi Normal University Xi'an China; ^2^ College of Life Sciences, Shaanxi Normal University Xi'an China; ^3^ River Basin Research Center Gifu University Gifu Japan

**Keywords:** mitogenome, *P. Cactorum* complex, phylogeny, *Phytophthora*

## Abstract

*Phytophthora* is a genus of oomycetes that includes many aggressive pathogens capable of devastating farmlands and forests worldwide. Among the oldest and most well‐known species, *P. cactorum* exhibits morphological and genetic similarities to other homothallic species within subclade 1a, which complicates the understanding of their evolutionary relationships. This study primarily compared seven *P. cactorum* strains from diverse origins with three closely related species in subclade 1a, utilizing mitogenome sequences for analysis. The circular mitogenomes of the four species were nearly identical in size and comprised 38 protein‐coding genes (PCGs), 25 transfer ribonucleic acid genes, and 2 ribosomal RNA genes. The mitochondrial genomes exhibited a higher percentage of A/T compared to G/C content. The majority of AT‐skew and GC‐skew values among the 38 PCGs were positive, with the AT‐skew demonstrating a more pronounced bias than the GC‐skew. The Ka/Ks ratios revealed that 35 PCGs underwent significant purifying selection. Although the AliGROOVE analysis indicated notable similarities among the subclade 1a species, four PCGs exhibited significantly higher pairing frequency compared to the complete mitogenome. The results from the phylogenetic analysis aligned with the pairwise genetic distances, indicating that *P. cactorum* is more closely related to *P. pseudotsugae* than to *P. hedraiandra*. Furthermore, we found that the *nad9* gene is informative to differentiate closely related *Phytophthora* species within subclade 1a, akin to the *cox1* gene.

## Graphical abstract

Mitogenomic analysis revealed the evolutionary relationships among Phytophthora 1a subclade species. Novel markers were identified to be sufficiently informative to distinguish between closely related species.

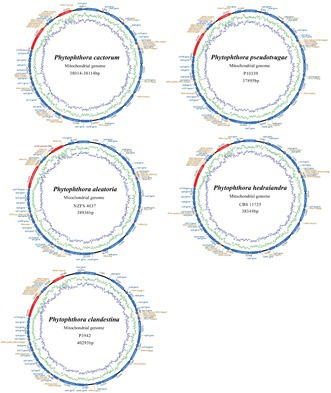

## 1 Introduction

*Phytophthora*, a genus of oomycetes, is recognized for its significant impact as an agricultural pathogen and its capacity to act as an invasive species in forest ecosystems.

(McGowan and Fitzpatrick 2020; Martin et al. 2012; O'Brien et al. 2009). To gain deeper insights into the genetic relationships within this genus, Martin et al. (2014) conducted a comprehensive analysis that scrutinized sequences from seven nuclear genes and four mitochondrial genes, encompassing a total of 90 formally designated taxa and 17 provisional taxa. The outcome of this extensive examination produced 11 clades based on phylogenies. Yang et al. (2017) further refined the phylogenetic understanding of *Phytophthora* by expanding their dataset to include 142 formally described *Phytophthora* species and 43 informally named species, thus significantly enhancing our knowledge in this area. The taxonomy of the genus *Phytophthora* has undergone substantial revision due to the recent study by Abad et al. (2023); however, issues surrounding species complexes remain challenging and may lead to confusion for researchers attempting accurate identification. Among these phylogenetic investigations (Robideau et al. 2011; Martin et al. 2014; Choi et al. 2015; Abad et al. 2023), several mitochondrial markers—including *cox1*, *cox2*, *nad9*, *rps10*, and *secY*—have been compared and demonstrated individual advantages in elucidating the evolutionary relationships of *Phytophthora* species. Notably, due to its high resolution and stable amplification, the *cox1* gene has gained widespread acceptance and adoption.

Among the *Phytophthora* species, *Phytophthora cactorum* (Lebert & Cohn) J. Schröt (1886) is one of the earliest and most well‐known members (Erwin and Ribeiro 1996). With a global distribution and a broad host range, it causes substantial economic losses through fruit rots and damage to orchard crowns (Marin et al. 2021; Sutton et al. 2014; Maas 1998). In 1983, researchers distinguished *P. pseudotsugae* from *P. cactorum*, based on subtle but consistently identifiable morphological traits (Hamm and Hansen 1983). Subsequently, *P. idaei* and *P. hedraiandra* were formally recognized as distinct species in 1995 and 2004, respectively (Kennedy and Duncan 1995; De Cock and Lévesque 2004). Later, within subclade 1a, *P. hedraiandra*, *P. aleatoria*, and *P. alpina* were assigned their taxonomic positions, along with hybrid species such as *P. ×serendipita* (*P. cactorum* × *P. hedraiandra*) and *P. ×pelgrandis* (*P. cactorum* × *P. nicotianae* ) (Man in't Veld et al. 2012; Scott et al. 2019; Bregant et al. 2020; Nirenberg et al. 2009). This body of research has significantly enhanced our comprehension of the *Phytophthora* genus and its diverse species.

Despite significant advancements in clarifying and distinguishing the *Phytophthora* phylogeny, the processes of coevolution and speciation within subclade 1a species remain highly complex. Bhat et al. (2006) conducted an extensive study in California, analyzing 132 isolates of *P. cactorum*. Their research revealed that 30.8% of the variance was attributed to the host plants, 24.5% to the geographical origins of the isolates, and 15% to the specific niche of the isolate. These results highlight the substantial role of both host plants and geographical factors in driving the speciation dynamics of subclade 1a species. In a more recent study, Nellist et al. (2021) used whole‐genome phylogenetic analysis to provide genomic evidence of distinct lineages within *P. cactorum*. This significant finding suggests that *P. cactorum* should be considered a species complex rather than a single, homogeneous species.

This issue is further complicated by the work of Bourret et al. (2022), who used sequences from three barcoding loci to conduct a comparative analysis of 30 *P. cactorum* strains and 12 *P. pseudotsugae* strains, all from California, alongside a comprehensive global collection of 112 conspecific strains. Their findings raised doubts about the classification of several isolates previously identified as *P. cactorum*, suggesting that these isolates may accurately belong to *P. hedraiandra* or *P. ×serendipita*. This observation highlights the ongoing challenges in accurately distinguishing between closely related *Phytophthora* species, despite the widespread use of both nuclear and mitochondrial DNA loci for previous studies (Martin et al. 2014; Yang et al. 2017; Blair et al. 2008). The significant morphological and genetic similarities among these species continue to pose a major obstacle to their reliable differentiation.

The mitochondrial genome has become a widely used molecular marker in fungal research due to its simple genetic structure, maternal inheritance, rapid evolutionary rate, high specificity, and ease of detection (Sandor et al. 2018; Aguileta et al. 2014). This has led to a significant increase in the routine sequencing of oomycetes mitogenomes in recent years, aimed at clarifying their evolutionary relationships (Winkworth et al. 2022, 2021; Seo et al. 2022; Yuan et al. 2017). Notably, 44 mitogenomes from the *Phytophthora* genus have been released, representing 11 clades, with two species—*P. cactorum* and *P. aleatoria*—included in subclade 1a (Winkworth et al. 2022). These mitogenomes, particularly those within clade 1, exhibit consistent size and gene order. However, interspecific length variations have been observed, ranging from 37,922 to 39,870 base pairs (bp).

Despite extensive studies on the *P. cactorum* mitogenome, significant knowledge gaps remain regarding the *P. cactorum* complex and the differentiation of closely related species, such as *P. hedraiandra* and *P. pseudotsugae* . Therefore, the primary aim of this study was to sequence the complete mitogenomes of multiple *P. cactorum* strains and the closely related species within subclade 1a. Through the analysis of these newly sequenced mitogenomes, we sought to clarify the evolutionary relationships among subclade 1a species and identify molecular markers for the precise identification of these closely related species. Additionally, our study aimed to provide insights into the evolutionary dynamics and patterns of mitogenomes within the *P. cactorum* complex.

## 2 Materials and Methods

### 2.1 Samples and DNA Extraction

This study used 40 strains of *Phytophthora* species, including seven *P. cactorum* strains with different hosts and geographical locations, and 33 strains of other 33 *Phytophthora* species, as well as one *Globisporangium ultimum* strain for the mitogenomic analysis (Table 1). The mitogenomes of 16 *Phytophthora* species including *P. asparagi* , *P. botryosa*, *P. cactorum*, *P. capensis* , *P. capsici*, *P. cinnamomi*, *P. citricola* , *P. citrophthora*, *P. clandestina* , *P. colocasiae*, *P. gonapodyides*, *P. hedraiandra*, *P. humicola* , *P. ilicis* , *P. plurivora*, and *P. pseudotsugae* , were obtained from local culture collections.

**TABLE 1:** Taxa used in this study.

Species	Clade^a^	Strain	GenBank accession no. of the mitogenomes	Hosts/habitat	Origins	References
*Phytophthora cactorum*	1a	CH98PA11	OK555490	*Paeonia albiflora*	Japan	This study
		cac_nanlinB	OK574285	NA	China	This study
		262HNP	OK574284	Forest soil	China	This study
		CH9812411	OK574295	*Paeonia albiflora*	Japan	This study
		CH98LOQ1	OK574294	*Eriobotrya janonica*	Japan	This study
		CH02MKPy001	OK574293	*Aralia elata*	Japan	This study
		10,300	BK011979	Strawberry	Norway	Winkworth et al. (2022)
*P. pseudotsugae*		P10339 ^(T)^	OK574304	*Pseudotsuga menziesii*	USA	This study
*P. hedraiandra*		CBS 111725 ^(T)^	OK574290	*Viburnum* sp.	Netherlands	This study
*P. aleatoria*		NZFS 4037	BK059193	*Pinus radiata*	New Zealand	Winkworth et al. (2022)
*P. clandestina*	1b	P3942	OK574302	*Trifolium subterraneum*	Australia	This study
*P. andina*	1c	CBS 122202^(T)^	NC_015619	Soil	Ecuador	Lassiter et al. (2015)
*P. infestans*		ATCC 16981	NC_002387	NA	NA	Lassiter et al. (2015)
*P. ipomoeae*		CBS 122203	NC_015622	*Ipomoea longipedunculata*	Mexico	Lassiter et al. (2015)
*P. mirabilis*		CBS 122204	NC_015606	*Mirabilis jalapa*	Mexico	Lassiter et al. (2015)
*P. phaseoli*		P11078	NC_015616	NA	Delaware	Lassiter et al. (2015)
*P. nicotianae*	1	JM1	NC_035725	Tobacco	China	Yuan et al. (2017)
*P. botryosa*	2a	CBS 581.69 ^(T)^	OK574288	*Hevea brasiliensis*	Malaysia	This study
*P. citrophthora*		CBS 950.87	OK574289	*Citrus* sp.	USA	This study
*P. colocasiae*		Fj20_1	OK574296	NA	China	This study
*P. capsici*	2b	Pc389	OK574305	Pepper	Taiwan	This study
*P. citricola*	2c	P0713	OK574299	NA	Argentina	This study
*P. capensis*		CBS 128319 ^(T)^	OK574291	*Curtisia dentata*	South Africa	This study
*P. plurivora*		CBS 124093 ^(T)^	OK574286	*Fagus sylvatica*	Germany	This study
*P. ilicis*	3	P3939	OK574301	*Ilex aquifolium*	Canada	This study
*P. pluvialis*		NZFS 3000	OK574297	*Pinus radiata*	New Zealand	Studholme et al. (2016)
*P. palmivora*	4	ICMP 17709	NC_056125	*Pachira aquatica*	NA	Winkworth et al. (2022)
*P. agathidicida*	5	ICMP 16471	MN883601	*Agathis australis*	New Zealand	Winkworth et al. (2021)
*P. cocois*		ICMP 16949	NC_056121	*Cocos nucifera*	Hawaii	Winkworth et al. (2020)
*P. heveae*		ICMP 19451	NC_056122	*Hevea brasiliensis*	Malaysia	Winkworth et al. (2020)
*P. humicola*	6a	P3826 ^(T)^	OK574300	*Citrus* sp.	Taiwan	This study
*P. gonapodyides*	6b	P7050	OK574303	NA	UK	This study
*P. asparagi*	6	CBS 132095	OK574292	*Asparagus officinalis*	USA	This study
*P. sojae*	7b	P6497	NC_009385	Soybean	USA	Martin et al. (2007)
*P. cinnamomi*	7c	CBS 144.22 ^(T)^	OK574287	*Cinnamomum burmannii*	Indonesia	This study
*P. sansomeana*	8a	ATCC MYA‐4455 ^(T)^	NC_045089	*Glycine* sp.	USA	Cai and Scofield (2020)
*P. ramorum*	8c	Pr‐102	NC_009384	NA	USA	Martin et al. (2007)
*P. polonica*	9a	NA	NC_029397	NA	NA	NA
*P. captiosa*	9b	ICMP 17567	NC_056123	*Eucalyptus saligna*	New Zealand	Winkworth et al. (2022)
*P. kernoviae*	10	NZFS 3630	OK574298	*Pinus radiata*	New Zealand	Studholme et al. (2016)
*Globisporangium ultimum*		CBS 805.95	NC_014280	Tobacco seedlings	Canada	Lévesque et al. (2010)

*Note:* International identification abbreviations: CBS, Westerdijk Fungal Biodiversity Institute; ICMP, International Collection of Microorganisms from Plants; NA, not available; NZFS, New Zealand Forest Research Institute Culture Collections; P, World *Phytophthora* Genetic Resource Collection; T, type strain.

^a^ Molecular phylogenetic clade according to Yang et al. (2017).

The mycelium was grown on V8 agar at 20°C for 5 days in the incubator, and one block of an actively growing hyphal region was transferred by a sterile wire hook and allowed to grow in V8 broth. Following incubation at 20°C for 3–5 days, genomic deoxyribonucleic acid (DNA) was extracted using the methodology provided by Kageyama et al. (2003) with modifications by Li et al. (2011) to include a magnetic bead purification stage (MagExtractor‐Plant Genome; Toyobo Co., Osaka, Japan). The integrity of DNA samples was evaluated using agarose gel electrophoresis and UV spectroscopy. The NanoDrop One spectrophotometer (Thermo Scientific, Wilmington, USA) and Qubit 3.0 Fluorometer were utilized to determine the DNA's purity and concentration (Invitrogen, Shanghai, China). High‐quality DNA samples that passed the internal QA/QC were frozen at −20°C for the next experiment.

### 2.2 Library Construction and High‐Throughput Sequencing

The high‐quality DNA samples were randomly fragmented using a Covaris Focused‐ultrasonicator, and the DNA libraries were produced using the Illumina DNA library preparation protocol. An Agilent 2100 Bioanalyzer (Agilent, California, USA) and a quantitative PCR machine were used to assess the library's integrity. The quality‐assured DNA library was sequenced using an Illumina NovaSeq 6000 instrument. The original sequences obtained by Illumina NovaSeq 6000 sequencing were screened for quality according to the following protocol: paired reads were removed when the N content of a sequencing read exceeded 10% of the number of read bases, when the proportion of sequencing reads containing low‐quality (Q ≤ 5) bases exceeded 50%, or when any sequencing read contained an adapter sequence.

### 2.3 Sequence Assembly, Annotation, and Analysis

After adapter removal and quality trimming, the high‐quality fragments were assembled by Reduced Complexity (ARC) software to obtain a complete circular mitochondrial genome. Then the protein‐coding regions (PCGs), transfer ribonucleic acids (tRNA), and ribosomal RNA genes (rRNA) were determined by aligning them with the reported mitochondrial genomes of *Phytophthora* species in Geneious v.11.1.2 software (Auckland, New Zealand) with MAFFT v. 7.5 using the Auto option (Katoh and Standley 2013). The complete mitogenomic DNA sequences of *P. asparagi* , *P. botryosa*, *P. cactorum*, *P. capensis* , *P. capsici*, *P. cinnamomi*, *P. citricola* , *P. citrophthora*, *P. clandestina* , *P. colocasiae*, *P. gonapodyides*, *P. hedraiandra*, *P. humicola* , *P. ilicis* , *P. kernoviae*, *P. plurivora*, *P. pluvialis* , and *P. pseudotsugae* were uploaded to the GenBank database (Table 1).

The mitochondrial gene structure maps, nucleotide composition, base composition, and sequence length were evaluated using Geneious v.11.1.2. Geneious was also used to remove all start and stop codons and extract 38 PCGs. Nucleotide bias was measured using the formulae for AT‐skew and GC‐skew: AT‐skew = (A − T)/(A + T) and GC‐skew = (G − C)/(G + C). Estimates of evolutionary divergence based on the PCGs of mitogenomes and nucleotide pair frequencies for the whole mitogenomes and each protein coding gene were computed by MEGA v.7.0.26. Besides, in MEGA v.7.0.26 software, relative synonymous codon use (RSCU) statistics were estimated using the formula (RSCUij = (Xij • ni)/Xij). GraphPad Prism 8.1 software generated a histogram and visual representation of codon use based on RSCU values. DnaSP v.5.0 was used to compute the rates of nonsynonymous substitutions (Ka) and synonymous substitutions (Ks) of each PCG (Barcelona, Spain).

AliGROOVE was used to evaluate the sequence divergence heterogeneities of the whole mitogenomic sequences. Indels in the dataset were treated as ambiguous. This metric established the pairwise sequence distance between individual terminals. The obtained scoring distance between sequences was then compared with similarity over the entire data matrix. Values varied from −1 (distances are very different from the average for the entire data matrix) to +1 (distances match the average for the entire matrix). This provided an indirect measure of the heterogeneity of a given sequence in comparison with the full dataset.

### 2.4 Phylogenetic Inference

To establish evolutionary relationships among *P. cactorum*, *P. pseudotsugae* , *P. hedraiandra*, and other closely related species, four mitogenomes, including *P. capsici*, *P. pluvialis* , *P. cinnamomi*, and *P. kernoviae*, were assembled and annotated from whole genomic DNA sequences downloaded from the GenBank database, and 18 complete mitogenomes, including *P. agathidicida*, *P. aleatoria*, *P. andina* , *P. cactorum*, *P. captiosa*, *P. cocois*, *P. heveae*, *P. infestans* , *P. ipomoeae* , *P. mirabilis* , *P. nicotianae* , *P. palmivora* , *P. phaseoli*, *P. polonica*, *P. ramorum, P. sansomeana*, *P. sojae*, and *Globisporangium ultimum* from the GenBank database were used (Table 1). Multiple sequence alignment and concatenation sequence evaluation were performed using PhyloSuite v.1.2.2 (Zhang et al. 2020).

The phylogenetic trees were generated both with a complete mitogenome dataset and the concatenated sequences of 33 PCGs, which coexisted in all tested species. *Globisporangium ultimum* (GenBank accession number: NC014280) was utilized as the outgroup to determine the phylogenetic tree's root. Bayesian Inference (BI) and Maximum Likelihood (ML) methods were performed, and ModelFinder v2.2.0 (Kalyaanamoorthy et al. 2017) was used to find the ideal model for nucleotide sequences using the BIC criterion. BI phylogenies for the complete mitogenome dataset were generated using MrBayes v3.2.7a (Ronquist et al. 2012) under the GTR + F + G4 model. The analysis comprised two parallel runs over 10,000,000 generations, with the initial 25% of sampled data discarded as burn‐in. ML phylogenies were inferred using IQ‐TREE v2.2.0 (Nguyen et al. 2015) under the GTR + R4 + F model, incorporating 10,000 ultrafast bootstraps (Minh et al. 2013) and the Shimodaira–Hasegawa–like approximate likelihood‐ratio test (Guindon et al. 2010). Similarly, for the PCG datasets, BI phylogenies were inferred using MrBayes v3.2.7a under a partition model, following the same protocol of two parallel runs for 10,000,000 generations with a 25% burn‐in. The ML phylogenies for these datasets were obtained using IQ‐TREE v2.2.0 under an Edge‐linked partition model, also utilizing 10,000 ultrafast bootstraps and the Shimodaira–Hasegawa–like test. Furthermore, for Bayesian inference, convergence diagnostics were assessed via the Estimated Sample Size (ESS) and the Potential Scale Reduction Factor (PSRF). An ESS value below 100 indicates potential under‐sampling of the parameter, while the PSRF should approach 1.0 as the runs converge.

## 3 Results

### 3.1 Genome Structure and Nucleotide Composition

**FIGURE 1:**
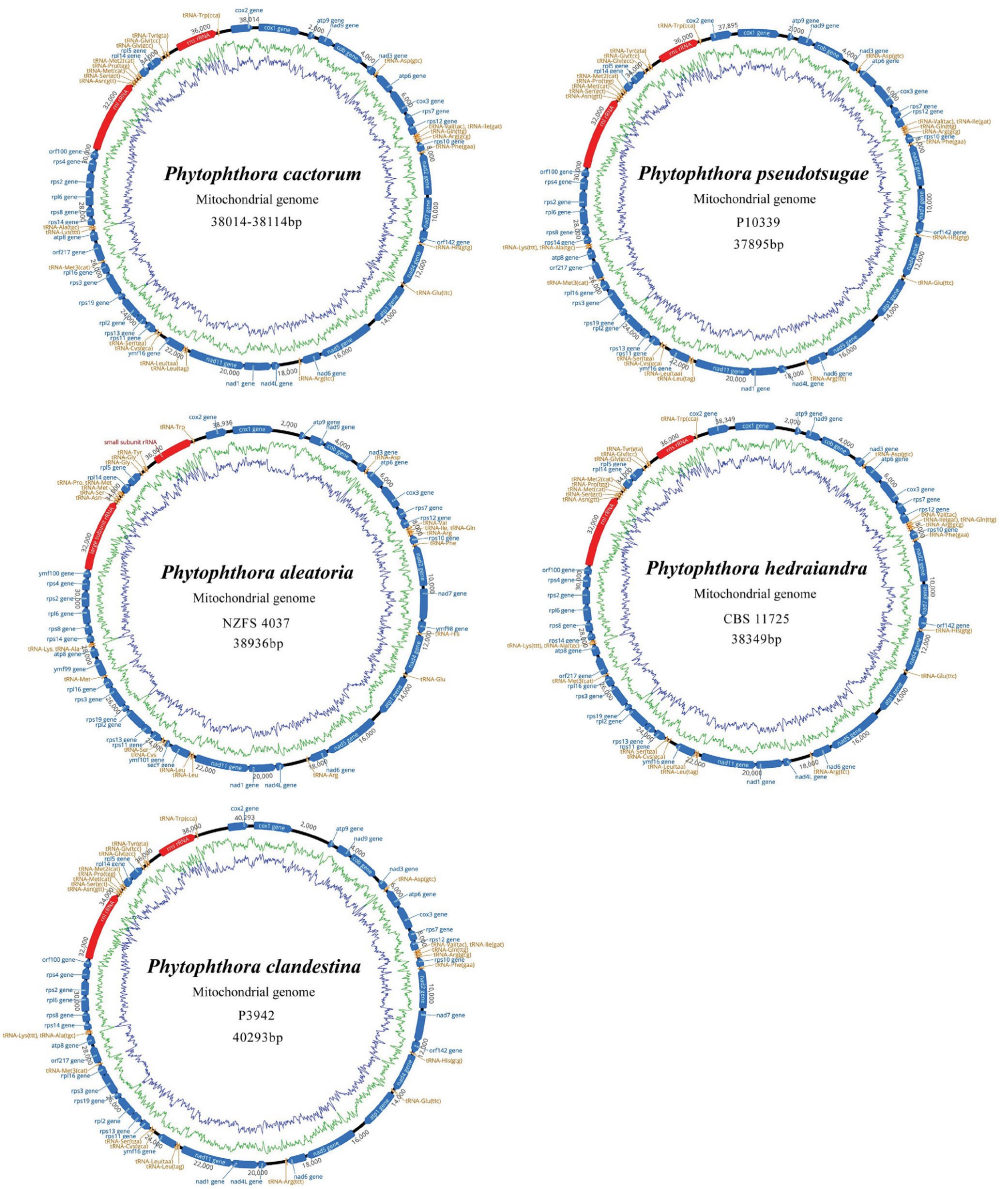
Circular maps of the mitogenome of *Phytophthora cactorum*, *P. pseudotsugae* , *P. aleatoria*, *P. hedraiandra*, and *P. clandestina* . The inner green and blue closed circular curves represent the contents of A + T and G + C.

We sequenced and assembled 23 complete mitochondrial genomes of 18 *Phytophthora* species in this study (Table 1). Five closely related *Phytophthora* species, including *P. cactorum*, *P. pseudotsugae* , *P. hedraiandra*, and *P. aleatoria*, which belong to subclade 1a, and *P. clandestina* , belonging to subclade 1b, were compared by the mitogenomic analysis. The mitogenome sizes of *P. cactorum*, including seven strains from different hosts and locations, ranged from 38,014 bp to 38,114 bp (Figure 1, Table S1). The mitogenome sequences of *P. pseudotsugae* , *P. hedraiandra*, *P. aleatoria*, and *P. clandestina* were 37,895, 38,349, 38,936, and 40,293 bp in length, respectively (Figure 1; Table S1). All nine mitochondrial genomes of these five species contained 38 PCGs, two rRNA genes, and 25 tRNA genes (Figure 1; Table S2). Without gene rearrangement, these species' gene structure and order were identical to other described *Phytophthora* mitogenomes. The light strand (L‐strand) encoded 12 PCGs (*atp6*, *cox3*, *nad1*, *nad2*, *nad3*, *nad4L*, *nad11*, *rps7*, *rps10*, *rps11*, *rps12*, and *rps13*) and nine tRNA genes (Asp, Val, Ile, Gln, Arg, Phe, Leu, and Ser), and other genes were located on the heavy strand (H‐strand) (Figure 1, Table S2).

All 11 mitochondrial genomes exhibited a substantial A/T bias in nucleotide composition compared to G + C content (A + T concentrations ranging from 78.23% to 79.01%), which is characteristic of oomycetes (Table S1). The GC content of each gene ranged from 8.1% to 51.4%, with the *tRNA‐Asp* gene of the *P. clandestina* strain P3942 having the highest value, and the *ymf16* genes of the *P. cactorum* strain CH98LOQ1 and *P. aleatoria* strain NZFS 4037 having the lowest values (Table S2).

### 3.2 RNA Genes and Protein‐Coding Genes

There were 25 tRNA genes observed in the mitogenomes of *P. cactorum*, *P. pseudotsugae* , *P. hedraiandra*, *P. aleatoria*, and *P. clandestina* that were between 71 and 91 bp in length (Table S2). Three tRNA genes, *tRNA‐Leu* (TAG, TAA), *tRNA‐Ser* (TGA, GCT), and *tRNA‐Gly* (GCC, TCC), were determined by two forms of anticodons, whereas a single type decided the others. The H‐strand contained small and large rDNA genes between 1,501 and 1,503 bp in length and 2,649 and 2,652 bp in length, respectively (Table S2). On average, the GC content of the rRNA genes was 34.5% in *P. cactorum*, 34.4% in *P. pseudotsugae* , 34.4% in *P. hedraiandra*, 34.3% in *P. aleatoria*, and 34.4% in *P. clandestina* , respectively (Table S2). The lengths of PCGs, tRNAs, and rRNAs for *P. cactorum* (CH98PA11, cac_nanlinB, 262HNP, CH9812411, CH98LOQ1, CH02MKPy001, and 10,300), *P. pseudotsugae* (P10339), *P. hedraiandra* (CBS111725), *P. aleatoria* (NZFS 4037) and *P. clandestina* (P3942) were compared, but no significant differences were found (Table S1).

**FIGURE 2:**
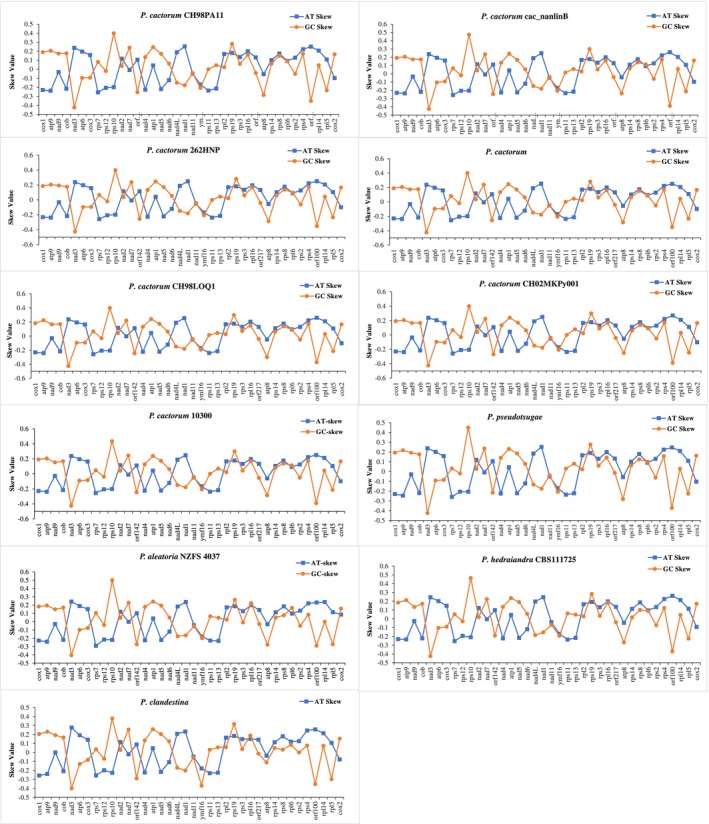
The AT/GC skew value of 38 PCGs for seven *Phytophthora cactorum* strains and four closely related species including *P. pseudotsugae* , *P. aleatoria*, *P. hedraiandra*, and *P. clandestina* .

The 38 PCGs of *P. cactorum*, *P. pseudotsugae* , *P. hedraiandra*, *P. aleatoria*, and *P. clandestina* were 27,867–28,080, 27,874–27,843, 28,038, and 27,832 bp in length, accounting for 73.19%–73.31%, 73.56%, 72.60%, 73.65%, and 69.07% of the whole mitochondrial genome (Table S1). All the PCGs started with the typical ATG codon and terminated with TAA, TAG, or TGA. The intergenic nucleotides, including interval sequences and overlapping sequences of adjacent genes, existed among all the PCGs except for the *cox2* gene (Table S2). The AT‐skew and GC‐skew of the four species were remarkably comparable. More than half of the AT‐skew and GC‐skew values of the 38 PCGs in the nine mitochondrial genomes were positive, indicating that bases A and G were more abundant than T and C (Figure 2). In most instances, the AT‐skew was more apparent than the GC‐skew (Figure 2). Nucleotide skew may be attributable to the mutation and selection pressure during replication and transcription. Ten PCGs, including *cox1*, *atp9*, *cob*, *nad3*, *rps10*, *nad4*, *nad5*, *nad4L*, *nad1*, and *rpl5*, exhibited larger fluctuations in the AT/GC‐skew value (Figure 2), indicating that the selection and mutational pressure on these genes may be significantly distinct from that of other genes.

### 3.3 Usage of Mitogenome Codon

**FIGURE 3:**
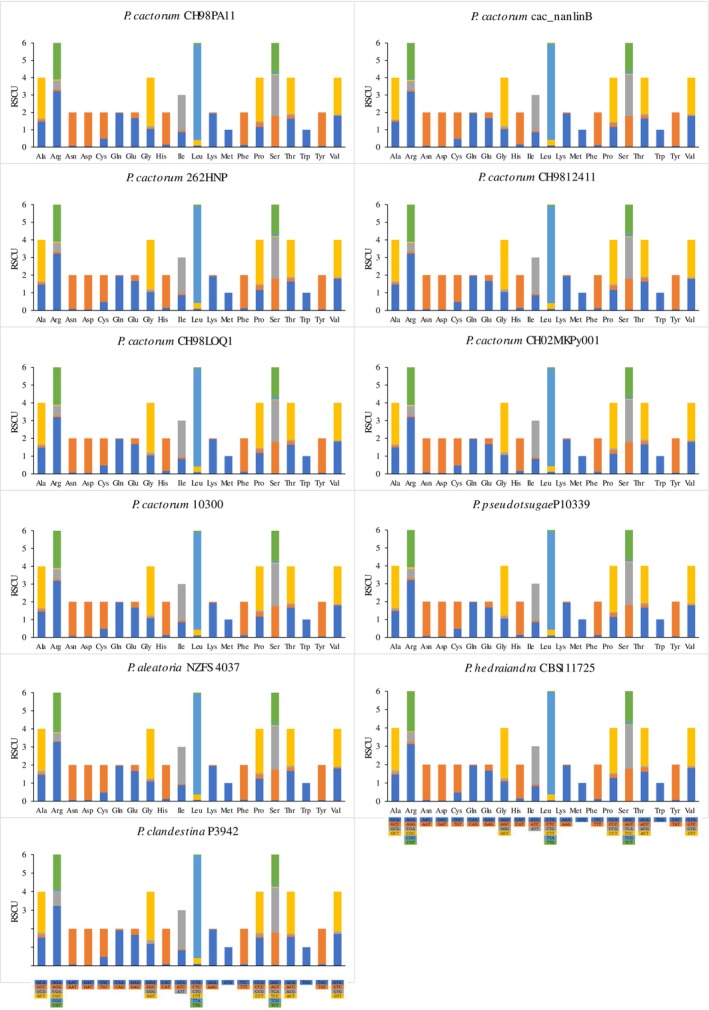
Relative synonymous codon usage (RSCU) of the mitogenomes of seven *Phytophthora cactorum* strains and four closely related species, including *P. pseudotsugae* , *P. aleatoria*, *P. hedraiandra*, and *P. clandestina* .

RSCU was implemented to evaluate mitochondrial gene codon use (Figure 3). When RSCU = 1, it represented that the frequency of usage of codons was identical to that of other degenerate codons; when RSCU > 1, it suggested that the codon was used more frequently. The RSCU value of most amino acids in the four species was not 1, indicating that amino acid use was biased to varying degrees (Figure 3). In all of the 11 mitogenomes, the amino acids Arg, Leu, and Ser employed six distinct codons and had the highest relative codon utilization values (Figure 3). Leu levels were the highest in the mitogenomes of *P. hedraiandra* and *P. aleatoria* (Figure 3). Met and Trp had the lowest levels of relative codon usage and were encoded by a single codon (Figure 3). Identical RSCU values for most amino acids of five species indicated that the gene function in the *Phytophthora* clade 1 was extremely similar.

### 3.4 Variations, Genetic Distance, and Evolution Rates of PCGs

The genetic distance could be used to assess the varying mutation pressures between genes. Calculations of pairwise genetic distances (*p*‐distance) revealed the conservation and divergence of PCG sequences among the four *Phytophthora* species (Table 2). Lower *p*‐distance values were found among different strains of *P. cactorum*, while higher values were found between *P. cactorum* and other closely related species (Table 2). However, the interspecific *p*‐distance between *P. pseudotsugae* and *P. cactorum* was quite similar to the intraspecific *p*‐distance of *P. cactorum*. In contrast, the interspecific *p*‐distance among *P. aleatoria*, *P. hedraiandra*, *P. clandestina*, and *P. cactorum* significantly increased (Table 2), indicating that *P. cactorum* and *P. pseudotsugae* have similar evolutionary divergence in the mitochondrial genome.

**TABLE 2:** Estimates of evolutionary divergence between the protein‐coding gene sequences of the mitogenomes of *P. cactorum*, *P. aleatoria*, *P. pseudotsugae* , *P. hedraiandra*, and *P. clandestina* .

		*P. cactorum*							*P. aleatoria*	*P. pseudotsugae*	*P. hedraiandra*	*P. clandestina*
		262HNP	cac_nanlinB	CH02MKPy001	CH98LOQ1	CH98PA11	CH9812411	10,300	NZFS 4037	P10339	CBS111725	P3942
*P. cactorum*	262HNP		0.0004	0.0003	0.0004	0.0002	0.0002	0.0003	0.0008	0.0006	0.0007	0.0014
	cac_nanlinB	0.0025		0.0003	0.0003	0.0003	0.0003	0.0004	0.0009	0.0005	0.0007	0.0014
	CH02MKPy001	0.0038	0.0035		0.0003	0.0002	0.0002	0.0004	0.0010	0.0004	0.0008	0.0016
	CH98LOQ1	0.0037	0.0035	0.0030		0.0003	0.0003	0.0003	0.0010	0.0005	0.0007	0.0015
	CH98PA11	0.0007	0.0024	0.0037	0.0036		0.0000	0.0002	0.0008	0.0005	0.0007	0.0013
	CH9812411	0.0007	0.0024	0.0037	0.0036	0.0000		0.0002	0.0008	0.0005	0.0007	0.0013
	10,300	0.0028	0.0027	0.0039	0.0038	0.0027	0.0027		0.0009	0.0004	0.0008	0.0015
*P. aleatoria*	NZFS 4037	0.0241	0.0239	0.0247	0.0249	0.0242	0.0242	0.0245		0.0010	0.0009	0.0014
*P. pseudotsugae*	P10339	0.0052	0.0051	0.0058	0.0058	0.0052	0.0052	0.0056	0.0249		0.0007	0.0014
*P. hedraiandra*	CBS111725	0.0175	0.0171	0.0179	0.0179	0.0174	0.0174	0.0176	0.0212	0.0187		0.0012
*P. clandestina*	P3942	0.0486	0.0482	0.0492	0.0492	0.0485	0.0485	0.0487	0.0521	0.0498	0.0473	

*Note:* The number of base differences per site between sequences is shown. Standard error estimate(s) are shown above the diagonal.

**FIGURE 4:**
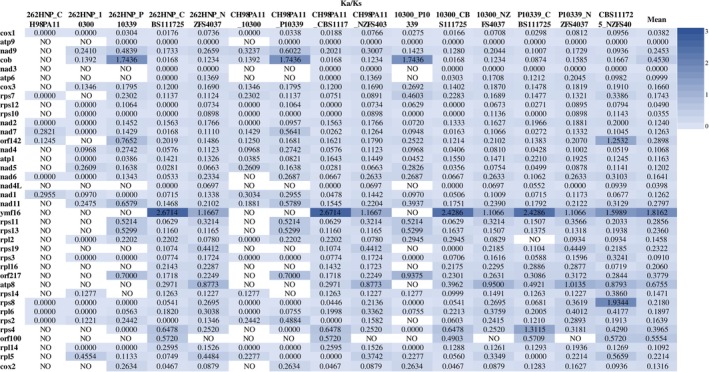
The rates of non‐synonymous substitutions and synonymous substitutions for each PCG in pairwise mitochondrial genomes of *P. cactorum* (CH98PA11, 262HNP, and 10,300), *P. pseudotsugae* (P10339), *P. hedraiandra* (CBS111725), and *P. aleatoria* (NZFS4037).

For further evaluation of the evolutionary relationships between these subclade 1a *Phytophthora* species, the rates of nonsynonymous substitution (Ka)/synonymous substitution (Ks) in pairwise mitochondrial genomes of *P. cactorum* (CH98PA11, 262HNP, and 10,300), *P. pseudotsugae* (P10339), *P. hedraiandra* (CBS111725), and *P. aleatoria* (NZFS 4037) were determined (Figure 4). Ka/Ks < 1, Ka/Ks = 1, and Ka/Ks > 1 indicated purifying selection, neutral mutation, and positive selection. Thirty four PCGs were under strong purifying selection with Ka/Ks values less than 1, whereas *cob*, *ymf16*, *atp8*, *rps8*, and *rps4* genes showed positive selection with Ka/Ks values greater than 1 in the pairwise mitogenomes of *P. cactorum*, *P. pseudotsugae* , *P. aleatoria*, and *P. hedraiandra* (Figure 4). Compared to other genes, the Ka/Ks values of *atp9* and *nad3* were zero, representing no mutation, indicating that their expression levels were the highest.

**FIGURE 5:**
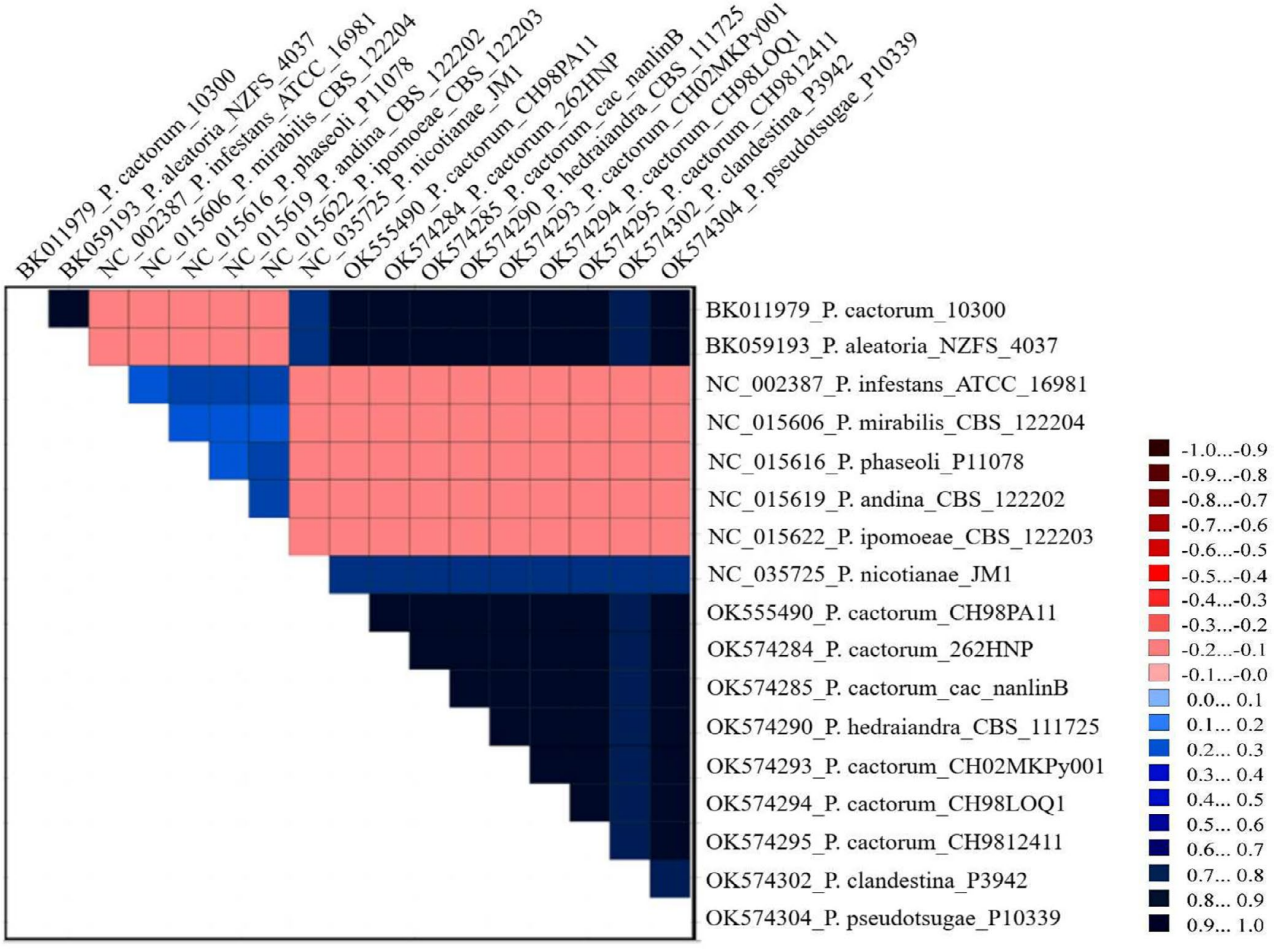
AliGROOVE analysis of the whole mitogenome sequences for 11 *Phytophthora* species of clade 1 considering their nucleotide composition. The obtained mean similarity score between sequences is represented by a colored square. The site scores are ranging from −1, indicating great difference in sequence composition (red coloring), to +1, indicating similarity to other sequence composition (blue coloring).

**FIGURE 6:**
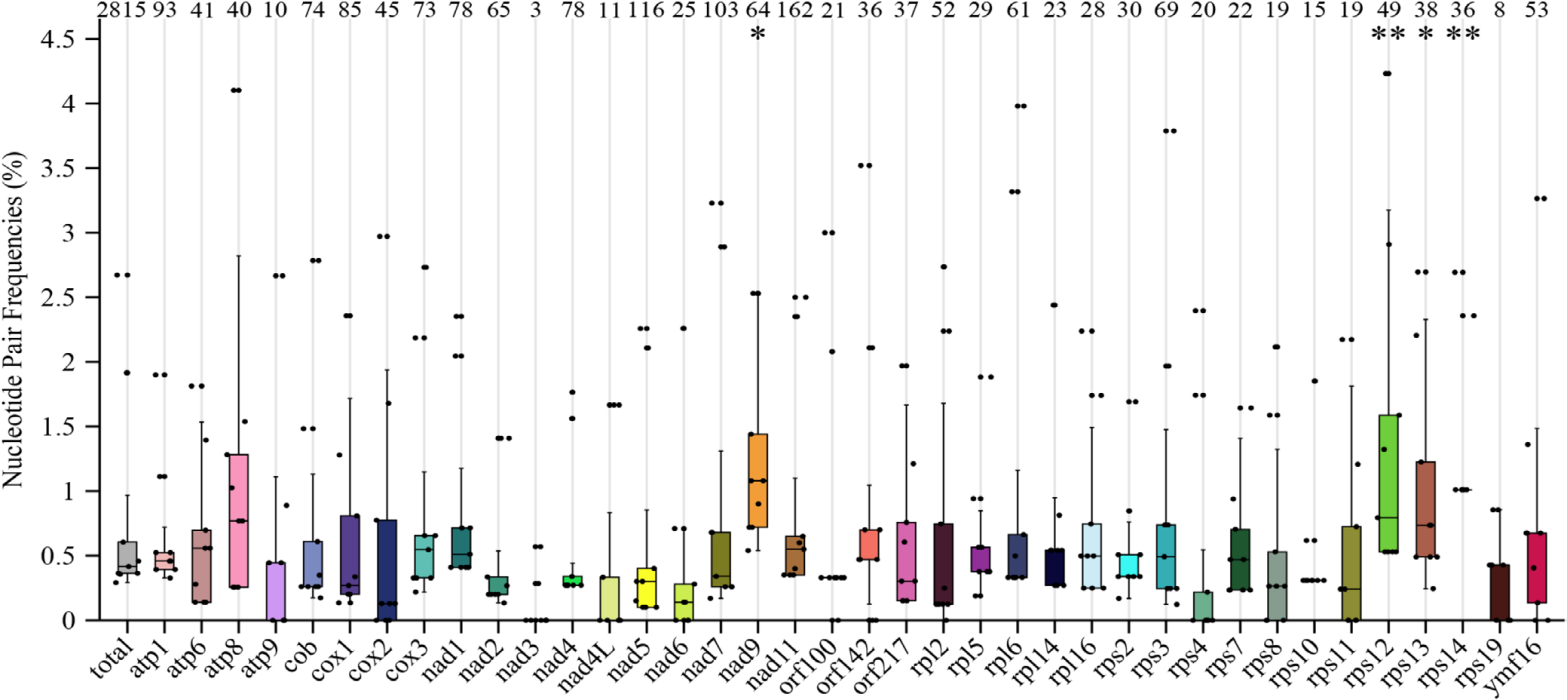
Analysis of nucleotide pair frequencies for the whole mitogenomes and each protein coding gene. Four closely related subclade 1a species and seven different *P. cactorum* strains were tested. The mutation sites including transitional pairs (Si) and transversional pairs (Sv) against *P. cactorum* strain 10,300 were computed and listed on the top of the columns. A paired sample *T*‐test was used to perform a differential analysis between each gene and the ‘total’. An asterisk indicates that the nucleotide pair frequency of that gene is significantly higher than the total: *p < 0.05; **p < 0.01.

The AliGROOVE analysis of the complete mitogenome sequence for 11 *Phytophthora* species within clade 1 revealed obvious variations in sequence composition among different subclades (Figure 5). Species belonging to subclade 1c, highlighted in red, demonstrated distinct differences compared to the other subclades, represented in blue. Within the blue cluster, *P. nicotianae* and *P. clandestina* (subclade 1b) displayed minor heterogeneities, while species in subclade 1a were relatively similar. To further investigate mutation levels in subclade 1a species, nucleotide pair frequencies for the complete mitogenomes and each PCG were analyzed against *P. cactorum* strain 10,300. Results indicated that four PCGs—*nad9*, *rps12*, *rps13*, and *rps14*—showed significantly higher mutation rates compared to the entire mitogenome, with the *nad9* gene exhibiting the most mutation sites (Figure 6).

### 3.5 Phylogenetic Analyses

**FIGURE 7:**
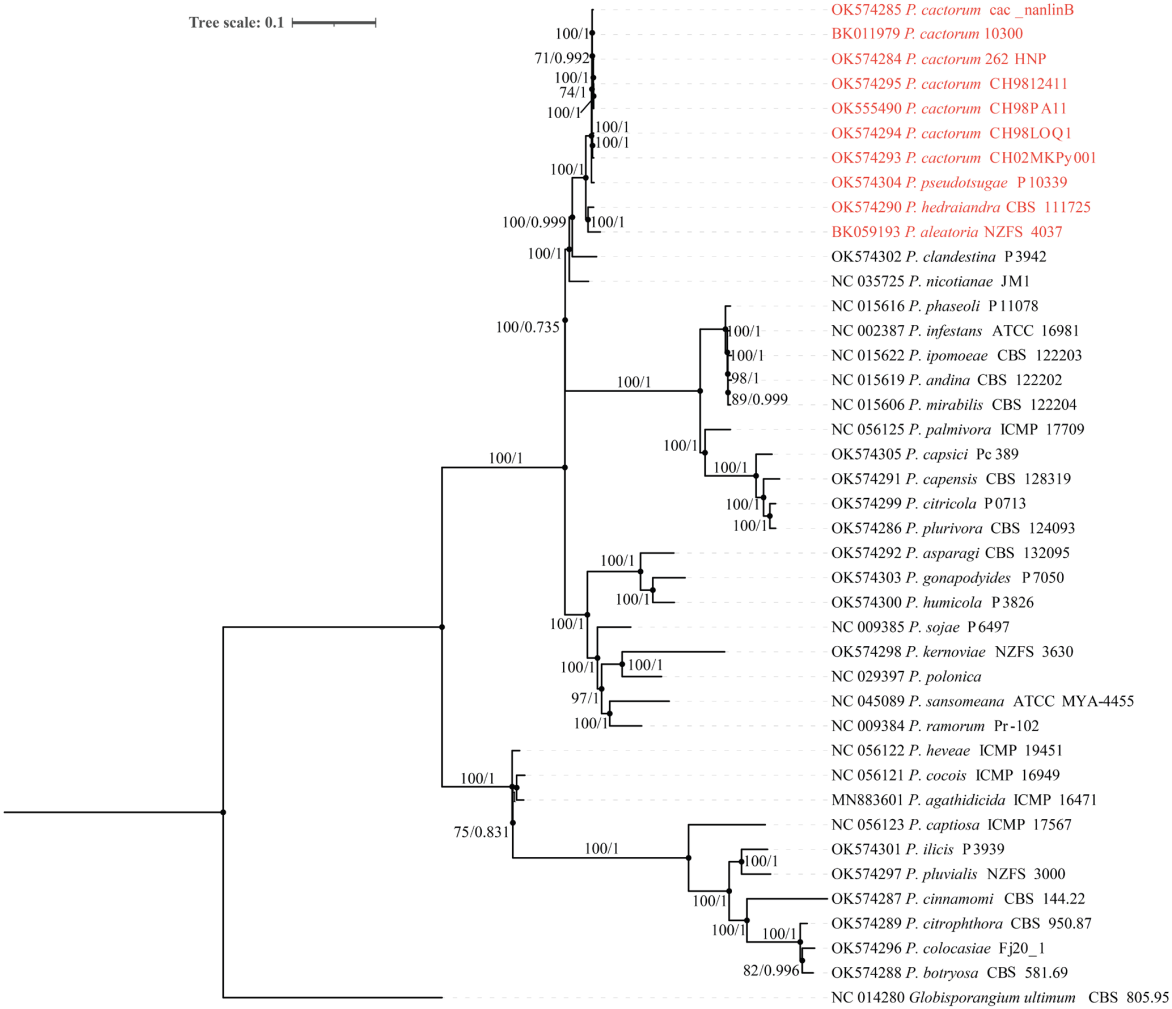
Phylogenetic tree of 41 *Phytophthora* strains constructed by Maximum Likelihood (ML) and Bayesian Inference (BI) methods based on the complete mitogenomes. *Globisporangium ultimum* was used as the outgroup. Numerals at nodes are ML bootstrap support values (left) and Bayesian posterior probabilities (right), respectively.

**FIGURE 8:**
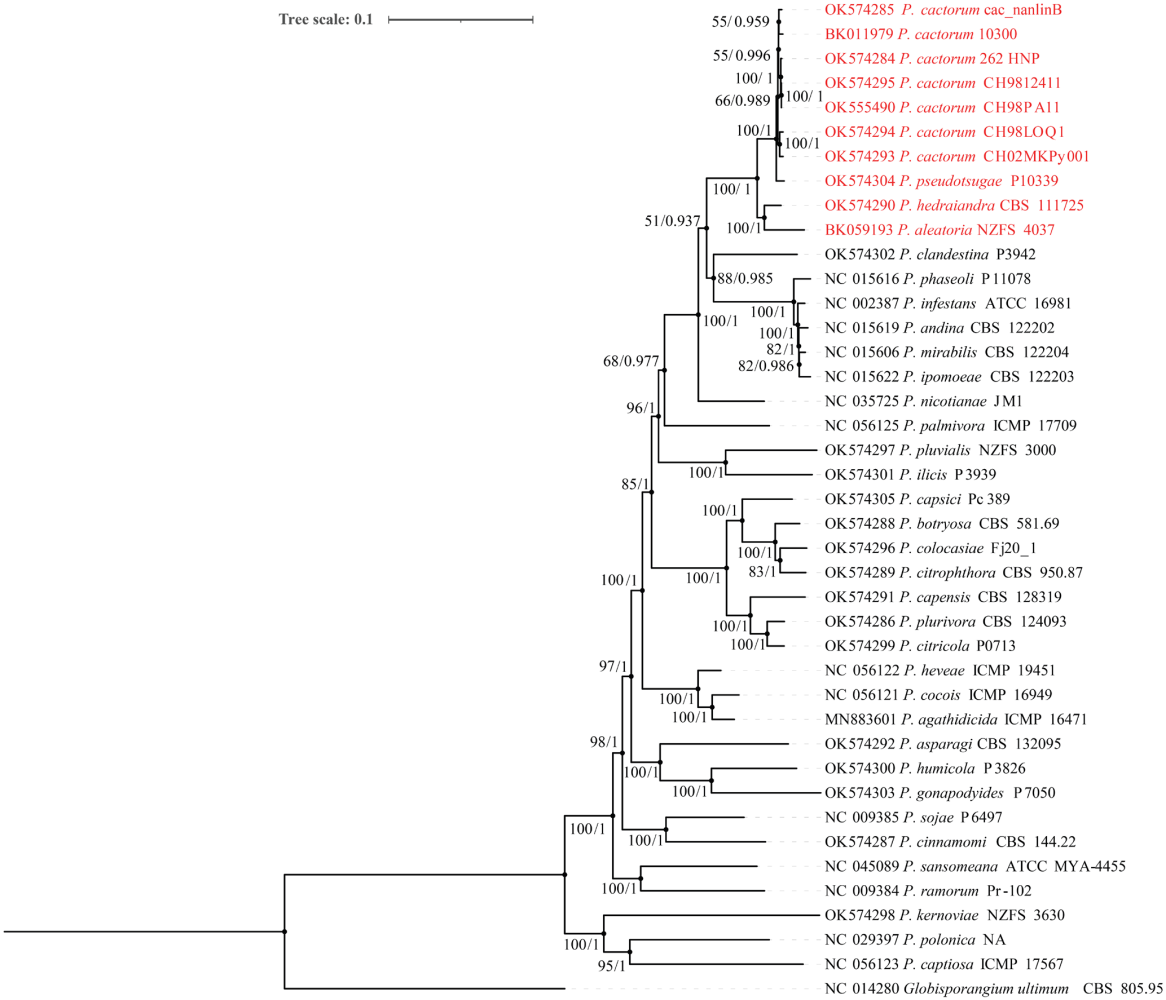
Phylogenetic tree of 41 *Phytophthora* strains constructed by Maximum Likelihood (ML) and Bayesian Inference (BI) methods based on the concatenated sequences of 33 PCGs. *Globisporangium ultimum* was used as the outgroup. Numerals at nodes are ML bootstrap support values (left) and Bayesian posterior probabilities (right), respectively.

The phylogenetic analysis contained 41 strains, including 34 *Phytophthora* species from 10 clades and one *Globisporangium* species as the out‐group species (Figures 7 and 8). For each species, the DNA sequences of 33 PCGs common to the genus *Phytophthora* and *Globisporangium* were concatenated. Phylogenetic trees constructed using Bayesian Inference and Maximum Likelihood yielded identical topologies, exhibiting high bootstrap and posterior probability values for both the complete mitogenomes and the PCGs. These results demonstrated that the species of subclade 1a clustered together and diverged from all other species with strong support values (100/1) in both the complete mitogenome and PCGs trees (Figures 7 and 8). Additionally, *P. pseudotsugae* clustered with all *P. cactorum* strains with high support values (100/1), forming a distinct clade, while *P. aleatoria* grouped with *P. hedraiandra* with similarly high support values (100/1), forming another separated subclade. Thus, it can be concluded that *P. pseudotsugae* is genetically closer to *P. cactorum* than *P. hedraiandra* and *P. aleatoria* in the mitogenome. Notably, there were significant topological differences between the complete mitogenome trees and the PCGs trees (Figures 7 and 8). The PCGs‐based trees reflected the same phylogenetic relationships as those reported in recent studies, whereas the complete mitogenome‐based trees differed, likely due to varied gene orders among the clades and subclades.

## 4 Discussion

Eleven mitogenomes including seven *P. cactorum* strains with different origins and four closely related species, *P. pseudotsugae* , *P. hedraiandra*, *P. aleatoria*, and *P. clandestina* , were compared. In terms of genome size, GC content, and gene content, the comparison revealed that *P. pseudotsugae* , *P. aleatoria*, and *P. hedraiandra* were very similar to *P. cactorum*. Nevertheless, all subclade 1a mitogenomes were smaller than the subclade 1b mitogenomes of *P. clandestina* . This result was consistent with the latest research on Peronosporaceae mitogenomes by Winkworth et al. (2022). Combined with the studies of Winkworth et al. (2022) and Yuan et al. (2017), *Phytophthora* mitogenomes were smaller than those of other related oomycete species in the Pythiales order (*Globisporangium ultimum*, 59,689 bp; *Py. insidiosum*, 54,989 bp) (Tangphatsornruang et al. 2016; Lévesque et al. 2010) and had a compact arrangement of genes with few noncoding nucleotides. Mitogenomes of *Phytophthora* subclade 1a samples typically included 38 PCGs, 2 rRNAs, and 25 tRNAs encoding 19 amino acids. Gene duplication events were not detected, showing that the mitochondrial gene components are highly conserved.

Prior research has speculated that while inverted repeats (IR) are a significant characteristic of early‐diverging Peronosporales mitogenomes, they are not characteristic of *Phytophthora* mitogenomes. Yuan et al. (2017) compared nine *Phytophthora* mitogenomes and found that only *P. ramorum* contained IR. Winkworth et al. (2022) found that 11 different *Phytophthora* species had IR longer than 150 bp in the mitogenome. However, of the 23 mitogenomes assembled in this study, including 18 different *Phytophthora* species, *P. asparagi* (clade 6) and *P. sojae* (subclade 7b) contained only direct repeats, *P. kernoviae* (clade 10) contained IR only, *P. cinnamomi* (subclade 7c) contained both, and others contained no repeats longer than 100 bp. Therefore, we agree with the observations of Yuan et al. (2017) and Winkworth et al. (2022) that large IR were lost early in the evolution of Peronosporaceae. We also suggest that smaller repeats were distributed more frequently, but with more variability, in different clades of *Phytophthora*. Although the cause of the IR loss in Peronosporaceae remains unknown, it is possible that the IR areas are not conserved and were lost during evolution due to diverse environmental selection pressures. Further investigations on the IR‐containing *Phytophthora* species with abundant hosts or ecological niches will help reveal this reason and in understanding the gene rearrangement in mitogenomes of *Phytophthora* as well (Martin et al. 2007).

In numerous clades of *Phytophthora* phylogeny, the closely related species commonly clustered into big subclades, leading to the establishment of species complexes, such as the “*P. cactorum* complex” in subclade 1a, the “ *P. citricola* complex” in subclade 2c, and the “*P. cryptogea* complex” in subclade 8a (Yang et al. 2017). Phylogenies based on nuclear sequences showed that *P. hedraiandra* clustered together with *P. cactorum*, forming a separate branch (Blair et al. 2008). The same results were also obtained by Yang et al. (2017). In their phylogeny, *P. cactorum* and *P. hedraiandra* clustered with strong support. However, in our study, the phylogeny based on 33 PCGs of the mitogenome showed *P. pseudotsugae* strain P10339 and seven *P. cactorum* strains with different origins clustered with high support values (100/1) (Figure 7, Figure 8), indicating *P. pseudotsugae* was closer to *P. cactroum* than to *P. hedraiandra*. These results were supported by Martin et al. (2014) in a prior study, where their phylogenies were based on concatenated sequences of mitochondrial loci only and a combination of nuclear and mitochondrial loci, and *P. hedraiandra* was positioned beneath *P. cactorum* and *P. pseudotsugae* in the cluster. Meanwhile, similar relations among *P. cactorum*, *P. pseudotsugae* , *P. hedraiandra*, and *P. aleatoria* were revealed in the phylogenetic analyses by Scott et al. (2019) based on the concatenated sequences of the ITS, *ß*‐tubulin and *cox1* regions, in which *P. pseudotsugae* was closer to *P. cactorum*, while *P. aleatoria* was closer to *P. hedraiandra*. *P. cactorum* and *P. pseudotsugae* were still identified as sister taxa in the most recent analysis by Abad et al. (2023), which used the *cox1* gene and six nuclear loci. Another point to note is that there are insufficient morphological evidences to clearly differentiate these species. De Cock and Lévesque (2004) cited the type of antheridia as a crucial morphological characteristic for differentiating *P. hedraiandra* from *P. cactorum*. According to their findings, the majority of *P. hedraiandra*'s antheridia should be sessile. However, Pánek et al. (2016) showed no statistically significant difference in antheridia sessility between *P. cactorum* and *P. hedraiandra*. Although other research has shown that *P. cactorum* and *P. hedraiandra* have very distinct single‐strand‐conformation polymorphism patterns (Gallegly and Hong 2008), no reliable evidence was found based on both AFLP genotyping and DNA sequence data as well as morphological and growth characters of *P. cactorum* species complex in Europe, to support *P. hedraiandra* as a distinct species (Pánek et al. 2016). The only convincing evidence probably was the hybrid from *P. hedraiandra* and *P. cactorum*. *P*. ×*serendipita*, generated by hybridization between *P. hedraiandra* and *P. cactorum*, differed from both parents by producing abortive oospores and distorted antheridia (Man in't Veld et al. 2012). Therefore, additional research on mating and reproductive capacity and genome‐scale phylogenetic analysis will be required to clarify the *P. cactorum* complex.

The *Phytophthora* genus causes several devastating plant diseases, is responsible for substantial economic losses to food crops and ornamentals (O'Brien et al. 2009), and is a grave danger to forest ecosystems (Jung et al. 2016). Several genetic markers, including the internal transcribed spacer (ITS) regions, the *β*‐tubulin gene, the elongation factor *1a* gene, the *tigA* fusion protein gene, the mitochondrial *cox* genes, the ras‐related protein gene *Ypt1*, and others, have been widely used to develop species‐specific detection tools (Li et al. 2022; Hieno et al. 2021; Dai et al. 2019; Yang and Hong 2018; Miles et al. 2017; Scibetta et al. 2012; Schena et al. 2006). The ITS sections and the mitochondrial *cox1*, *cox2*, and *rps10* genes were suggested as DNA barcodes for oomycetes (Foster et al. 2022; Choi et al. 2015; Robideau et al. 2011). Our findings indicate that the *nad9* and *cob* genes possess significant informative sites (Table S2), exhibit higher GC content (Table S1), display greater AT and GC‐skew values (Figure 2), and demonstrate larger Ka and Ks rates (Figure 4). The genes *nad9*, *rps12*, *rps13*, and *rps14* showed significantly higher nucleotide pair mutation frequencies compared to the complete mitogenome (Figure 6). Consequently, these genes may provide valuable insights for differentiating closely related species within subclade 1a. However, comparisons among 11 highly similar mitogenomes of *Phytophthora* clade 1 species revealed that nearly all tested single genes were unable to separate subclade 1a species from one another, with the exception of the *cox1* gene (Figures S1–S8). The *cox1* gene effectively distinguished four subclade 1a species (Figure S1). The *cox2* gene grouped the subclade 1a species into two clusters (Figure S2), whereas the *rps10* gene failed to separate them at all (Figure S3). The *cob*, *rps12*, *rps13*, and *rps14* genes exhibited a behavior similar to that of the *cox2* gene (Figures S2–S8). Although the *nad9* gene showed resolutions comparable to those of the *cox1* gene, it provided higher resolution among inter‐ and intra‐species of subclade 1a (Figure S4), suggesting that *nad9* could serve as a potential alternative gene for differentiating closely related *Phytophthora* subclade 1a species. This conclusion was supported by the study of Miles et al. (2017), in which the *nad9* locus and the intergenic spacer on the 5′ end have been the targets of considerable diagnostic research. Besides, it is important to consider the hybrids of closely related species, as these hybrids are often unstable evolutionary lineages, representing transient hybrid clones (Burgess 2015). This instability may pose challenges in discriminating hybrids using mitogenomic DNA markers. Therefore, integrating mitochondrial genes with nuclear genes will be crucial for clarifying the evolutionary status of these hybrid species.

## 5 Conclusions

In this study, we sequenced and assembled 23 complete mitochondrial genomes from 18 different *Phytophthora* species. We compared and analyzed seven *P. cactorum* strains of varied origins along with three other species from subclade 1a that are part of the *P. cactorum* complex. Our findings indicate that *P. cactorum* shares a closer evolutionary relationship with *P. pseudotsugae* than with *P. hedraiandra*, despite the relatively similar sizes, nucleotide compositions, gene arrangements, and codon usages observed among the *P. cactorum* complex species. Furthermore, the *nad9* gene demonstrated significant potential for distinguishing these closely related *Phytophthora* species. Overall, this work provides valuable insights into the *P. cactorum* complex, and the recommended DNA marker will offer new perspectives in the study of the *Phytophthora* genus and facilitate accurate metabarcoding of the *Phytophthora* community.

## Author Contributions

**Tao Lin:** conceptualization (equal), data curation (equal), formal analysis (equal), writing – original draft (equal). **Huiqin Wang:** conceptualization (equal), data curation (equal), formal analysis (equal), writing – original draft (equal). **Haiting Ma:** data curation (equal), formal analysis (equal), investigation (equal). **Jiaying Duan:** data curation (equal), formal analysis (equal), investigation (equal). **Wenxin Wang:** validation (equal). **Xiaoyu Shi:** formal analysis (equal), visualization (equal). **Yaling Li:** formal analysis (equal), visualization (equal). **Zengqiang Qian:** methodology (equal), software (equal), visualization (equal). **Nian Liu:** methodology (equal), software (equal), visualization (equal). **Jiabin Zou:** methodology (equal), software (equal), visualization (equal). **Ayaka Hieno:** resources (equal). **Koji Kageyama:** resources (equal). **Mingzhu Li:** funding acquisition (lead), project administration (lead), supervision (lead), writing – review and editing (equal).

## Conflicts of Interest

The authors declare no conflicts of interest.

## Supporting information


Data S1.

## Data Availability

The mitogenomes of *Phytophthora* strains sequenced and assembled in this study have been deposited in NCBI with accession numbers (Table 1). The datasets for phylogenetic analysis were deposited in the Mendeley Data database (https://data.mendeley.com/datasets/mh4k929nbw/2).
