# Supplementary material for: Evolutionary Relationships of the Phytophthora 1a Subclade Species Based on Complete Mitogenomes, and Novel Markers for Their Differentiation
Source: Ecol Evol. 2025 Mar 13;15(3):e71105. doi: 10.1002/ece3.71105 (PMC11906254; doi:10.1002/ece3.71105)
Supplement: Supplementary file 1 — Data S1. [file ECE3-15-e71105-s001.pdf]

TABlE S1 Base composition for protein-coding, tRNA and rRNA genes of the mitogenomes for *P. cactorum* , *P. pseudotsugae* , *P.aleatoria* , *P. hedraiandra* , and *P. clandestina.*

Species	Isolates	Bases	Complete genome	Protein-coding genes	tRNA	rRNA
	CH98PA11	A	14873	10752	560	1483
		G	4532	3188	414	858
		T	14867	11397	615	1253
		C	3744	2560	311	559
		A+T	29740	22149	1175	2736
		Total	38016	27867	1900	4153
	cac_nanlinB	A	14882	10755	556	1478
		G	4535	3184	413	858
		T	14910	11408	603	1256
		C	3744	2562	309	561
		A+T	29792	22163	1159	2734
		Total	38071	27879	1881	4153
	262HNP	A	14873	10753	554	1484
		G	4534	3191	415	858
		T	14864	11392	603	1252
		C	3743	2561	309	559
		A+T	29737	22145	1157	2736
		Total	38014	27867	1881	4153
P. cactorum	CH9812411	A	14873	10752	555	1483
		G	4532	3188	414	559
		T	14867	11397	603	1253
		C	3744	2560	309	559
		A+T	29740	22149	1158	2736
		Total	38016	27867	1881	4153
	CH98LOQ1	A	14899	10773	555	1478
		G	4535	3185	414	857
		T	14927	11403	602	1256
		C	3753	2566	310	561
		A+T	29826	22176	1157	2734
		Total	38114	27897	1881	4152
	CH02MKPy0	A	14904	10771	556	1481
		G	4534	3183	414	856
		T	14906	11403	602	1256
		C	3747	2564	309	560
		A+T	29810	22174	1158	2737
		Total	38091	27891	1881	4153
	10300	A	14877	11036	566	1481
		G	4538	3088	368	858
		T	14909	11282	592	1252
		C	3743	2673	355	562
		A+T	29787	22319	1158	2733
		Total	38068	28080	1881	4153
P. pseudotsug	P10339	A	14824	10767	555	1478
		G	4523	3185	414	858
		T	14822	11419	603	1259
		C	3726	2559	309	557
		A+T	29646	22186	1158	2737
		Total	37895	27874	1881	4152
		A	15376	11092	569	1486

		G	4486	3010	365	850
P.aleatoria	NZFS 4037	T	15387	11335	600	1260
		C	3687	2601	347	556
		A+T	30763	22427	1169	2746
		Total	38936	28038	1881	4152
		A	15054	10754	556	1478
		G	4517	3173	413	855
P. hedraiandr	CBS111725	T	15014	11392	604	1260
		C	3764	2554	308	559
		A+T	30068	22146	1160	2738
		Total	38349	27843	1881	4152
		A	15925	10798	551	1480
		G	4683	3184	414	853
P. clandestinc	P3942	T	15775	11395	608	1262
		C	3910	2508	310	558
		A+T	31700	22193	1159	2742
		Total	40293	27832	1883	4153

TABlE S2 Summary of gene feature of ten *Phytophthora* mitogenomes. [a]

Gene	Strand	Position Start/End										
		P. cactorum							*pseudotsug*	*P. aleatoria*	*hedraiand*	*. clandestina*
		CH98PA11	ac_nanlin	262HNP	CH9812411	CH98LOQ1	H02MKPy0	10300	P10339	NZFS 4037	CBS11172	P3942
cox1	H	1-1488	1-1488	1-1488	1-1488	1-1488	1-1488	1-1488	1-1488	1-1488	1-1488	1-1479
atp9	H	1818-2045	1815-2042	1817-2044	1818-2045	1819-2046	1820-2047	1819-2046	1787-2014	2481-2708	2186-2413	2946-3173
nad9	H	2213-2773	2210-2767	2212-2772	2213-2773	2240-2797	2216-2773	2213-2770	2181-2738	2874-3431	2592-3149	3337-3894
cob	H	2830-3981	2828-3979	2829-3980	2830-3981	2858-4009	2834-3985	2831-3982	2799-3950	3487-4638	3205-4353	3950-5101
nad3	L	4187-4540	4185-4538	4186-4539	4187-4540	4214-4567	4191-4544	4188-4541	4155-4508	4824-5177	4560-4913	5318-5671
tRNA-Asp	L	4578-4653	4577-4650	4578-4651	4579-4652	4606-4679	4583-4656	4580-4653	4547-4620	5216-5289	4952-5025	5707-5780
atp6	L	4675-5394	4673-5392	4674-5393	4675-5394	4702-5421	4679-5398	4676-5395	4643-5362	5312-6031	5048-5767	5816-6535
cox3	L	5420-6337	5418-6335	5419-6336	5420-6337	5447-6364	5424-6341	5421-6338	5388-6305	6068-6985	5804-6721	6560-7477
rps7	L	6397-6825	6395-6823	6396-6824	6397-6825	6424-6852	6401-6829	6398-6826	6365-6793	7043-7471	6779-7207	7533-7961
rps12	L	6800-7180	6798-7178	6799-7179	6800-7180	6827-7207	6804-7184	6801-7181	6768-7148	7446-7826	7182-7562	7936-8316
tRNA-Val	L	7198-7270	7196-7268	7197-7269	7198-7270	7226-7298	7204-7276	7199-7271	7166-7238	7845-7917	7580-7652	8333-8405
tRNA-Ile	L	7273-7346	7271-7344	7272-7345	7273-7346	7301-7374	7279-7352	7274-7347	7241-7314	7920-7993	7655-7728	8409-8482
tRNA-Gln	L	7347-7420	7346-7417	7347-7418	7348-7419	7376-7447	7354-7425	7349-7420	7316-7387	7995-8066	7730-7801	8484-8555
tRNA-Arg	L	7429-7504	7428-7501	7429-7502	7430-7503	7458-7531	7436-7509	7431-7504	7398-7471	8077-8150	7812-7885	8567-8640
rps10	L	7508-7834	7506-7832	7507-7833	7508-7834	7536-7862	7514-7840	7509-7835	7476-7802	8155-8481	7890-8216	8645-8971
tRNA-Phe	L	7853-7926	7851-7924	7852-7925	7853-7926	7881-7954	7859-7932	7854-7927	7821-7894	8500-8573	8235-8308	8990-9063
nad2	L	7933-9426	7931-9424	7932-9425	7933-9426	7961-9454	7939-9432	7934-9427	7901-9394	8580-1007	8315-9808	9070-10563
nad7	H	9542-1072	9540-1071	9541-1071	9542-1072	9570-1074	9548-1072	9543-1072	9510-1068	0189-1136	9924-1110	0679-11857
orf142	H	0731-1115	0730-1115	0730-1115	0731-1115	0759-1118	0738-1116	0732-1116	0699-1112	1377-1180	1114-1154	1870-12298
tRNA-His	H	1163-1123	1162-1123	1162-1123	1163-1123	1191-1126	1170-1124	1164-1123	1131-1120	1809-1188	1546-1161	2303-12374
nad4	H	1260-1273	1259-1273	1259-1273	1260-1273	1288-1276	1267-1274	1260-1273	1228-1270	1908-1338	1643-1311	2401-13876
tRNA-Glu	H	2766-1283	2765-1283	2765-1283	2766-1283	2794-1286	2773-1284	2766-1283	2734-1280	3414-1348	3149-1322	3901-13972
atp1	H	2918-1444	2917-1444	2917-1444	2918-1444	2946-1447	2925-1445	2918-1444	2886-1441	3566-1509	3301-1483	4052-15581
nad5	H	4742-1673	4743-1673	4741-1673	4742-1673	4770-1676	4747-1674	4743-1673	4704-1669	5524-1751	5074-1706	6139-18133
nad6	H	6780-1749	6781-1749	6779-1748	6780-1749	6808-1751	6785-1749	6781-1749	6742-1745	7562-1827	7112-1782	8175-18885
tRNA-Arg	H	7522-1759	7517-1758	7515-1758	7522-1759	7544-1761	7521-1759	7517-1758	7472-1754	8298-1837	7840-1791	8910-18982
nad4L	L	8244-1854	8239-1854	8236-1853	8244-1854	8266-1856	8243-1854	8238-1854	8194-1849	9191-1949	8580-1888	9667-19969
nad1	L	8549-1952	8544-1952	8541-1952	8549-1952	8571-1955	8548-1952	8543-1952	8499-1947	9496-2047	8885-1986	9973-20953
nad11	L	9526-2153	9521-2152	9518-2152	9526-2153	9548-2155	9525-2153	9520-2152	9476-2148	0473-2247	9862-2186	0950-22956
tRNA-Leu	L	1637-2172	1633-2171	1630-2171	1638-2172	1660-2174	1637-2171	1632-2171	1588-2167	2563-2264	1972-2205	3060-23144
tRNA-Leu	L	1731-2181	1726-2180	1723-2180	1731-2181	1753-2183	1730-2181	1725-2180	1681-2176	2657-2274	2065-2214	3155-23238
ymf16	H	1838-2258	1833-2259	1830-2257	1838-2258	1860-2262	1837-2259	1832-2257	1788-2254	2763-2350	2172-2290	3261-24004

tRNA-Cys	H	2800-2287	2785-2285	2792-2286	2800-2287	2812-2288	2789-2285	2789-2285	2740-2281	3719-2378	3154-2322	4212-24282
tRNA-Ser	L	2882-2296	2867-2295	2874-2295	2882-2296	2894-2297	2871-2295	2871-2295	2822-2290	3801-2388	3236-2332	4294-24378
rps11	L	2985-2340	2970-2338	2977-2339	2985-2340	2997-2341	2974-2339	2974-2339	2925-2334	3904-2432	3339-2375	4397-24813
rps13	L	3414-2382	3399-2380	3406-2381	3414-2382	3426-2383	3403-2381	3403-2381	3354-2376	4333-2474	3768-2417	4826-25242
rpl2	H	3853-2465	3838-2464	3845-2465	3853-2465	3865-2467	3842-2464	3842-2464	3793-2459	4775-2558	4207-2501	5271-26074
rps19	H	4663-2489	4648-2488	4655-2489	4663-2489	4675-2491	4652-2488	4652-2488	4603-2486	5585-2582	5017-2525	6078-26311
rps3	H	4903-2572	4888-2570	4895-2571	4903-2572	4915-2573	4892-2570	4892-2570	4844-2565	5825-2661	5257-2607	6315-27142
rpl16	H	5730-2613	5712-2611	5722-2612	5730-2613	5739-2614	5716-2612	5716-2612	5668-2607	6628-2703	6081-2648	7120-27524
tRNA-Met3	H	6137-2621	6119-2619	6129-2620	6137-2621	6146-2621	6123-2619	6123-2619	6075-2614	7035-2710	6488-2656	7527-27600
orf217	H	6226-2688	6208-2687	6218-2688	6226-2688	6235-2689	6212-2687	6212-2687	6164-2682	7124-2778	6577-2723	7623-28285
atp8	H	6950-2734	6932-2732	6942-2733	6950-2734	6959-2735	6936-2732	6936-2732	6888-2728	7848-2824	7301-2769	8347-28739
tRNA-Lys	H	7363-2743	7346-2741	7356-2742	7364-2743	7373-2744	7350-2742	7350-2742	7302-2737	8250-2832	7715-2778	8761-28833
tRNA-Ala	H	7440-2751	7422-2749	7432-2750	7440-2751	7449-2752	7426-2749	7426-2749	7378-2745	8326-2839	7791-2786	8837-28909
rps14	H	7531-2783	7513-2781	7523-2782	7531-2783	7540-2783	7517-2781	7517-2781	7469-2776	8417-2871	7882-2818	8928-29227
rps8	H	7841-2822	7823-2820	7833-2821	7841-2822	7850-2823	7827-2820	7827-2820	7779-2815	8725-2910	8190-2857	9236-29616
rpl6	H	8230-2883	8212-2881	8222-2882	8230-2883	8239-2884	8216-2882	8216-2882	8168-2877	9114-2971	8578-2918	9623-30228
rps2	H	8842-2943	8824-2941	8841-2943	8842-2943	8851-2944	8828-2942	8828-2942	8780-2937	9726-3031	9190-2978	0235-30828
rps4	H	9444-2990	9426-2988	9443-2990	9444-2990	9453-2991	9430-2989	9430-2989	9382-2984	0328-3078	9792-3025	0837-31298
orf100	H	9915-3022	9897-3020	9914-3022	9915-3022	9924-3025	9901-3022	9901-3020	9853-3015	0799-3108	0263-3056	1301-31603
rnl rRNA	H	0252-3290	0234-3288	0251-3290	0252-3290	0279-3292	0250-3289	0229-3287	0181-3282	1101-3375	0591-3323	1629-34280
tRNA-Asn	H	2907-3298	2890-3296	2907-3297	2908-3297	2934-3300	2906-3297	2884-3295	2836-3290	3757-3382	3246-3331	4287-34358
tRNA-Ser	H	2991-3308	2974-3306	2991-3307	2992-3308	3018-3310	2990-3307	2968-3305	2920-3300	3841-3392	3330-3341	4371-34459
tRNA-Met	H	3101-3317	3083-3315	3100-3317	3101-3317	3127-3319	3099-3317	3077-3314	3029-3310	3950-3402	3439-3351	4479-34550
tRNA-Pro	H	3216-3329	3198-3327	3215-3328	3216-3329	3242-3331	3214-3328	3192-3326	3144-3321	4065-3413	3554-3362	4593-34667
tRNA-Met2	H	3304-3337	3286-3335	3303-3337	3304-3337	3330-3340	3302-3337	3280-3335	3232-3330	4153-3422	3642-3371	4681-34752
rpl14	H	3393-3376	3375-3374	3392-3376	3393-3376	3419-3379	3391-3376	3369-3374	3321-3369	4242-3461	3731-3410	4770-35141
rpl5	H	3771-3430	3753-3428	3770-3430	3771-3430	3797-3433	3769-3430	3747-3428	3699-3423	4620-3515	4109-3464	5148-35681
tRNA-Gly	H	4311-3438	4294-3436	4311-3438	4312-3438	4338-3440	4310-3438	4288-3435	4240-3431	5161-3523	4650-3472	5689-35760
tRNA-Gly	H	4478-3455	4460-3453	4477-3454	4479-3455	4504-3457	4476-3454	4461-3253	4406-3447	5327-3539	4816-3488	5863-35934
tRNA-Tyr	H	4569-3465	4545-3462	4567-3465	4569-3465	4594-3467	4561-3464	4551-3463	4506-3458	5417-3550	4901-3498	5940-36023
rns rRNA	H	5014-3651	4990-3649	5012-3651	5014-3651	5042-3654	5016-3651	4997-3649	4952-3645	5886-3738	5351-3685	6550-38050
tRNA-Trp	H	6550-3662	6538-3660	6548-3661	6550-3662	6578-3664	6552-3662	6532-3660	6500-3657	7421-3749	6887-3695	8084-38155
cox2	H	7045-3782	7102-3787	7043-3781	7045-3782	7146-3792	7120-3789	7097-3787	6925-3770	7952-3872	7372-3814	9319-40095

Size (bp)										
			P. cactorum				*pseudotsug*	*P. aleatoria*	*hedraiand*	*. clandestina*
CH98PA11	cac_nanlinE	262HNP	CH98124111	CH98LOQ1	H02MKPy0	10300	P10339	NZFS 4037	CBS11172	P3942
1488	1488	1488	1488	1488	1488	1488	1488	1488	1488	1479
228	228	228	228	228	228	228	228	228	228	228
561	558	561	561	558	558	558	558	558	558	558
1152	1152	1152	1152	1152	1152	1152	1152	1152	1149	1152
354	354	354	354	354	354	354	354	354	354	354
76	74	74	74	74	74	74	74	74	74	74
720	720	720	720	720	720	720	720	720	720	720
918	918	918	918	918	918	918	918	918	918	918
429	429	429	429	429	429	429	429	429	429	429
381	381	381	381	381	381	381	381	381	381	381
73	73	73	73	73	73	73	73	73	73	73
74	74	74	74	74	74	74	74	74	74	74
74	72	72	72	72	72	72	72	72	72	72
76	74	74	74	74	74	74	74	74	74	74
327	327	327	327	327	327	327	327	327	327	327
74	74	74	74	74	74	74	74	74	74	74
1494	1494	1494	1494	1494	1494	1494	1494	1494	1494	1494
1179	1179	1179	1179	1179	1179	1179	1179	1179	1179	1179
429	429	429	429	429	429	429	429	429	429	429
73	72	72	72	72	72	72	72	72	72	72
1476	1476	1476	1476	1476	1476	1476	1476	1476	1476	1476
72	72	72	72	72	72	72	72	72	72	72
1530	1530	1530	1530	1530	1530	1530	1530	1530	1530	1530
1995	1995	1995	1995	1995	1995	1995	1995	1995	1995	1995
711	711	711	711	711	711	711	711	711	711	711
73	73	73	73	73	73	73	73	73	73	73
303	303	303	303	303	303	303	303	303	303	303
981	981	981	981	981	981	981	981	981	981	981
2007	2007	2007	2007	2007	2007	2007	2007	2007	2007	2007
85	83	83	83	83	83	83	83	83	83	85
84	84	84	84	84	84	84	84	84	84	84
744	762	744	744	762	762	744	762	738	738	744

71	71	71	71	71	71	71	71	71	71	71
85	85	85	85	85	85	85	85	85	85	85
417	417	417	417	417	417	417	417	417	417	417
411	411	411	411	411	411	411	411	414	411	417
807	807	807	807	807	807	807	807	807	807	804
237	237	237	237	237	237	237	267	237	237	234
819	816	819	819	816	816	816	816	795	816	828
405	405	405	405	405	405	405	405	405	405	405
74	74	74	74	74	74	74	74	74	74	74
663	663	663	663	663	663	663	663	663	663	663
393	393	393	393	393	393	393	393	393	393	393
75	73	73	73	73	73	73	73	73	73	73
74	73	73	73	73	73	73	73	73	73	73
300	300	300	300	300	300	300	300	300	300	300
381	381	381	381	381	381	381	381	381	381	381
606	606	606	606	606	606	606	606	606	606	606
594	594	594	594	594	594	594	594	594	594	594
462	462	462	462	462	462	462	462	462	462	462
312	312	312	312	330	324	303	303	291	303	303
2650	2650	2650	2650	2649	2650	2651	2649	2650	2649	2652
74	72	72	72	72	72	72	72	72	72	72
91	89	89	89	89	89	89	89	89	89	89
72	72	72	72	72	72	72	72	72	72	72
75	75	75	75	75	75	75	75	75	75	75
72	72	72	72	72	72	72	72	72	72	72
372	372	372	372	372	372	372	372	372	372	372
534	534	534	534	534	534	534	534	534	534	534
74	72	72	72	72	72	72	72	72	72	72
73	72	72	72	72	72	72	72	72	72	72
84	84	84	84	84	84	84	84	84	84	84
1503	1503	1503	1503	1503	1503	1502	1503	1502	1503	1501
72	72	72	72	72	72	72	72	72	72	72
777	777	777	777	777	777	777	777	777	777	777

GC Percentage										
			P. cactorum				*pseudotsug*	*P. aleatoria*	*hedraiand*	*. clandestina*
CH98PA11	:ac_nanlinE	262HNP	CH9812411	CH98LOQ1	H02MKPy0	10300	P10339	NZFS 4037	CBS11172	P3942
29.5%	29.4%	29.6%	29.5%	29.6%	29.6%	29.4%	29.2%	29.1%	29.5%	29.6%
29.8%	29.8%	29.8%	29.8%	29.4%	29.8%	29.8%	30.3%	29.4%	28.9%	28.5%
19.4%	19.5%	19.4%	19.4%	19.4%	19.4%	19.9%	19.5%	18.1%	18.6%	18.6%
26.0%	25.9%	26.0%	26.0%	26.0%	25.9%	25.9%	26.1%	25.1%	26.0%	25.6%
20.6%	20.6%	20.6%	20.6%	20.6%	20.6%	20.6%	20.6%	20.9%	20.6%	22.6%
47.4%	50.0%	50.0%	48.6%	50.0%	50.0%	50.0%	50.0%	50.0%	50.0%	51.4%
21.8%	21.4%	21.8%	21.8%	21.8%	21.8%	21.7%	21.4%	21.3%	21.4%	21.7%
25.7%	25.7%	25.7%	25.7%	25.7%	25.6%	25.9%	25.7%	25.3%	26.4%	26.4%
14.2%	14.0%	14.0%	14.2%	14.0%	14.0%	14.2%	14.5%	13.5%	13.8%	13.5%
27.3%	27.3%	27.3%	27.3%	26.5%	27.0%	27.3%	27.3%	25.7%	27.0%	26.0%
35.6%	34.2%	35.6%	35.6%	35.6%	35.6%	35.6%	35.6%	35.6%	35.6%	34.2%
41.9%	41.9%	41.9%	41.9%	41.9%	41.9%	41.9%	41.9%	40.5%	41.9%	43.2%
39.2%	40.3%	40.3%	40.3%	40.3%	40.3%	40.3%	40.3%	40.3%	40.3%	40.3%
30.3%	31.1%	31.1%	31.1%	31.1%	31.1%	31.1%	31.1%	31.1%	31.1%	32.4%
12.2%	11.6%	21.8%	12.2%	12.2%	12.2%	11.9%	12.2%	12.2%	12.5%	13.8%
43.2%	43.2%	43.2%	43.2%	43.2%	43.2%	43.2%	43.2%	43.2%	43.2%	41.9%
14.9%	14.8%	14.9%	14.9%	14.9%	14.9%	14.8%	14.7%	14.6%	14.7%	14.7%
27.2%	27.3%	27.4%	27.2%	27.2%	27.1%	27.1%	27.3%	26.4%	27.6%	27.6%
11.9%	12.4%	11.9%	11.9%	12.4%	12.1%	12.4%	11.9%	10.3%	12.1%	10.5%
41.1%	40.3%	40.3%	40.3%	40.3%	40.3%	40.3%	40.3%	38.9%	40.3%	40.3%
21.6%	21.7%	21.6%	21.6%	21.5%	21.5%	21.7%	21.7%	21.1%	21.5%	21.1%
41.7%	41.7%	41.7%	41.7%	41.7%	41.7%	41.7%	41.7%	41.7%	40.3%	41.7%
28.4%	28.5%	28.5%	28.4%	28.6%	28.6%	28.5%	28.4%	28.6%	28.8%	28.6%
22.8%	22.8%	22.8%	22.8%	22.8%	23.0%	22.8%	22.7%	22.0%	22.8%	22.6%
17.7%	17.9%	17.9%	17.7%	17.7%	17.7%	17.7%	17.7%	17.4%	17.6%	17.0%
34.2%	17.8%	34.2%	34.2%	34.2%	34.2%	34.2%	34.2%	32.9%	32.9%	31.5%
17.8%	34.2%	17.8%	17.8%	17.8%	17.8%	17.8%	17.5%	16.8%	17.8%	17.5%
26.5%	26.4%	26.6%	26.5%	26.4%	26.5%	26.3%	26.7%	26.6%	26.5%	25.3%
18.7%	18.7%	18.6%	18.7%	18.8%	18.7%	18.7%	18.6%	18.1%	18.1%	18.7%
34.1%	34.9%	34.9%	34.9%	34.9%	34.9%	34.9%	33.7%	33.7%	34.9%	35.3%
35.7%	35.7%	35.7%	35.7%	35.7%	35.7%	35.7%	35.7%	34.5%	35.7%	35.7%
8.5%	8.3%	8.5%	8.5%	8.1%	8.3%	8.5%	8.3%	8.1%	8.4%	9.4%

39.4%	38.0%	39.4%	39.4%	38.0%	38.0%	38.0%	38.0%	35.2%	39.4%	38.0%
41.2%	41.2%	41.2%	41.2%	42.4%	41.2%	41.2%	41.2%	41.2%	41.2%	40.0%
15.8%	16.1%	15.8%	15.8%	16.1%	15.8%	15.8%	15.8%	14.9%	15.8%	15.8%
21.4%	21.2%	21.4%	21.4%	21.9%	21.2%	20.9%	21.2%	20.8%	20.4%	21.3%
27.1%	27.0%	27.1%	27.1%	27.0%	27.1%	27.1%	27.1%	26.6%	27.3%	25.9%
16.5%	16.9%	16.5%	16.5%	16.9%	16.9%	16.9%	17.6%	16.0%	16.5%	16.2%
14.0%	14.0%	14.0%	14.0%	13.7%	13.7%	14.1%	14.3%	13.7%	14.2%	13.3%
24.7%	24.7%	24.4%	24.7%	24.9%	24.9%	24.9%	24.2%	25.4%	25.4%	26.2%
39.2%	39.2%	39.2%	39.2%	39.2%	39.2%	39.2%	39.2%	39.2%	39.2%	39.2%
11.3%	11.3%	11.3%	11.3%	11.3%	11.0%	11.2%	11.3%	10.6%	11.6%	11.0%
10.7%	10.7%	10.7%	10.7%	10.2%	10.2%	10.7%	9.9%	9.2%	10.4%	9.2%
42.7%	43.8%	43.8%	43.8%	43.8%	43.8%	43.8%	43.8%	43.8%	43.8%	43.8%
36.5%	35.6%	35.6%	35.6%	35.6%	35.6%	35.6%	35.6%	32.9%	35.6%	35.6%
21.3%	21.3%	21.3%	21.3%	22.0%	22.0%	21.0%	21.3%	20.3%	20.3%	20.3%
17.3%	17.1%	17.1%	17.3%	17.1%	17.1%	17.1%	16.8%	17.1%	18.1%	16.8%
14.9%	14.5%	15.0%	14.9%	14.5%	14.5%	14.5%	14.7%	11.9%	13.0%	13.7%
13.5%	13.6%	13.6%	13.5%	13.8%	13.8%	13.5%	13.6%	13.5%	13.8%	14.1%
12.6%	12.6%	12.6%	12.6%	12.6%	12.6%	12.6%	12.3%	12.8%	12.3%	14.1%
11.9%	11.5%	11.9%	11.9%	10.6%	11.1%	11.9%	11.6%	10.7%	11.6%	11.2%
33.3%	33.3%	33.3%	33.3%	33.3%	33.2%	33.3%	33.3%	32.9%	33.3%	33.2%
36.5%	37.5%	37.5%	37.5%	37.5%	37.5%	37.5%	38.9%	37.5%	37.5%	38.9%
39.6%	40.4%	40.4%	40.4%	40.4%	40.4%	40.4%	40.4%	39.3%	40.4%	39.3%
36.1%	36.1%	36.1%	36.1%	36.1%	36.1%	36.1%	36.1%	36.1%	36.1%	36.1%
42.7%	42.7%	42.7%	42.7%	42.7%	42.7%	42.7%	42.7%	42.7%	42.7%	42.7%
37.5%	37.5%	37.5%	37.5%	37.5%	37.5%	37.5%	37.5%	37.5%	37.5%	38.9%
18.0%	18.3%	18.0%	18.0%	18.3%	18.3%	18.0%	18.3%	18.3%	18.0%	18.0%
11.2%	11.4%	11.2%	11.2%	11.4%	11.4%	11.4%	11.6%	10.3%	11.6%	10.7%
37.8%	38.9%	38.9%	38.9%	38.9%	38.9%	38.9%	38.9%	38.9%	38.9%	38.9%
30.1%	30.6%	30.6%	30.6%	30.6%	30.6%	30.6%	30.6%	30.6%	30.6%	31.9%
39.3%	39.3%	39.3%	39.3%	39.3%	39.3%	39.3%	39.3%	39.3%	39.3%	40.5%
35.6%	35.7%	35.6%	35.6%	35.7%	35.7%	35.8%	35.5%	35.6%	35.5%	35.4%
30.6%	30.6%	30.6%	30.6%	30.6%	30.6%	30.6%	30.6%	29.2%	29.2%	29.2%
27.8%	27.9%	27.8%	27.8%	27.8%	27.8%	27.8%	27.7%	27.8%	27.9%	27.4%

Codon Start/Stop										
			P. cactorum				*pseudotsug*	*P. aleatoria*	*hedraiandı*	*. clandestina*
CH98PA11:	cac_nanlinE	262HNP	CH9812411	CH98LOQ1	H02MKPy0	10300	P10339	NZFS 4037	CBS11172:	P3942
ATG/TAA	ATG/TAA	ATG/TAA	ATG/TAA	ATG/TAA	ATG/TAA	ATG/TAA	ATG/TAA	ATG/TAA	ATG/TAA	ATG/TAA
ATG/TAA	ATG/TAA	ATG/TAA	ATG/TAA	ATG/TAA	ATG/TAA	ATG/TAA	ATG/TAA	ATG/TAA	ATG/TAA	ATG/TAA
ATG/TAA	ATG/TAA	ATG/TAA	ATG/TAA	ATG/TAA	ATG/TAA	ATG/TAA	ATG/TAA	ATG/TAA	ATG/TAA	ATG/TAA
ATG/TAA	ATG/TAA	ATG/TAA	ATG/TAA	ATG/TAA	ATG/TAA	ATG/TAA	ATG/TAA	ATG/TAA	ATG/TAA	ATG/TAA
ATG/TAA	ATG/TAA	ATG/TAA	ATG/TAA	ATG/TAA	ATG/TAA	ATG/TAA	ATG/TAA	ATG/TAA	ATG/TAA	ATG/TAG
ATG/TAA	ATG/TAA	ATG/TAA	ATG/TAA	ATG/TAA	ATG/TAA	ATG/TAA	ATG/TAA	ATG/TAA	ATG/TAA	ATG/TAA
ATG/TAA	ATG/TAA	ATG/TAA	ATG/TAA	ATG/TAA	ATG/TAA	ATG/TAA	ATG/TAA	ATG/TAA	ATG/TAA	ATG/TAA
ATG/TAA	ATG/TAA	ATG/TAA	ATG/TAA	ATG/TAA	ATG/TAA	ATG/TAA	ATG/TAA	ATG/TAA	ATG/TAA	ATG/TAA
ATG/TAA	ATG/TAA	ATG/TAA	ATG/TAA	ATG/TAA	ATG/TAA	ATG/TAA	ATG/TAA	ATG/TAA	ATG/TAA	ATG/TAA
ATG/TAA	ATG/TAA	ATG/TAA	ATG/TAA	ATG/TAA	ATG/TAA	ATG/TAA	ATG/TAA	ATG/TAA	ATG/TAA	ATG/TAA
ATG/TAA	ATG/TAA	ATG/TAA	ATG/TAA	ATG/TAA	ATG/TAA	ATG/TAA	ATG/TAA	ATG/TAA	ATG/TAA	ATG/TAA
ATG/TAA	ATG/TAA	ATG/TAA	ATG/TAA	ATG/TAA	ATG/TAA	ATG/TAA	ATG/TAA	ATG/TAA	ATG/TAA	ATG/TAA
ATG/TAA	ATG/TAA	ATG/TAA	ATG/TAA	ATG/TAA	ATG/TAA	ATG/TAA	ATG/TAA	ATG/TAA	ATG/TAA	ATG/TAA
ATG/TAA	ATG/TAA	ATG/TAA	ATG/TAA	ATG/TAA	ATG/TAA	ATG/TAA	ATG/TAA	ATG/TAA	ATG/TAA	ATG/TAA
ATG/TAA	ATG/TAA	ATG/TAA	ATG/TAA	ATG/TAA	ATG/TAA	ATG/TAA	ATG/TAA	ATG/TAA	ATG/TAA	ATG/TAA
ATG/TAA	ATG/TAA	ATG/TAA	ATG/TAA	ATG/TAA	ATG/TAA	ATG/TAA	ATG/TAA	ATG/TAA	ATG/TAA	ATG/TAA
ATG/TAA	ATG/TAA	ATG/TAA	ATG/TAA	ATG/TAA	ATG/TAA	ATG/TAA	ATG/TAA	ATG/TAA	ATG/TAA	ATG/TAA
ATG/TAA	ATG/TAA	ATG/TAA	ATG/TAA	ATG/TAA	ATG/TAA	ATG/TAA	ATG/TAA	ATG/TAA	ATG/TAA	ATG/TAA
ATG/TAA	ATG/TAA	ATG/TAA	ATG/TAA	ATG/TAA	ATG/TAA	ATG/TAA	ATG/TAA	ATG/TAA	ATG/TAA	ATG/TAA
ATG/TGA	ATG/TGA	ATG/TGA	ATG/TGA	ATG/TGA	ATG/TGA	ATG/TGA	ATG/TGA	ATG/TGA	ATG/TGA	ATG/TGA
ATG/TAA	ATG/TAA	ATG/TAA	ATG/TAA	ATG/TAA	ATG/TAA	ATG/TAA	ATG/TAA	ATG/TAA	ATG/TAA	ATG/TAA

ATG/TAA ATG/TAA ATG/TAA ATG/TAA ATG/TAA ATG/TAA ATG/TAA ATG/TAA ATG/TAA ATG/TAA ATG/TAA
ATG/TAA ATG/TAA ATG/TAA ATG/TAA ATG/TAA ATG/TAA ATG/TAA ATG/TAA ATG/TAA ATG/TAA ATG/TAA
ATG/TAA ATG/TAA ATG/TAA ATG/TAA ATG/TAA ATG/TAA ATG/TAA ATG/TAA ATG/TAA ATG/TAA ATG/TAA
ATG/TAA ATG/TAA ATG/TAA ATG/TAA ATG/TAA ATG/TAA ATG/TAA ATG/TAG ATG/TAG ATG/TAA ATG/TAA
ATG/TAA ATG/TAA ATG/TAA ATG/TAA ATG/TAA ATG/TAA ATG/TAA ATG/TAA ATG/TAA ATG/TAA ATG/TAA
ATG/TAA ATG/TAA ATG/TAA ATG/TAA ATG/TAA ATG/TAA ATG/TAA ATG/TAA ATG/TAA ATG/TAA ATG/TAA

ATG/TAA ATG/TAA ATG/TAA ATG/TAA ATG/TAA ATG/TAA ATG/TAA ATG/TAA ATG/TAA ATG/TAA ATG/TAA
ATG/TAA ATG/TAA ATG/TAA ATG/TAA ATG/TAA ATG/TAA ATG/TAA ATG/TAA ATG/TAA ATG/TAA ATG/TAA

ATG/TAA ATG/TAA ATG/TAA ATG/TAA ATG/TAA ATG/TAA ATG/TAA ATG/TAA ATG/TAA ATG/TAA ATG/TAA
ATG/TAA ATG/TAA ATG/TAA ATG/TAA ATG/TAA ATG/TAA ATG/TAA ATG/TAA ATG/TAA ATG/TAA ATG/TAA
ATG/TAA ATG/TAA ATG/TAA ATG/TAA ATG/TAA ATG/TAA ATG/TAA ATG/TAA ATG/TAA ATG/TAA ATG/TAA
ATG/TAA ATG/TAA ATG/TAA ATG/TAA ATG/TAA ATG/TAA ATG/TAA ATG/TAA ATG/TAA ATG/TAA ATG/TAA
ATG/TAA ATG/TAA ATG/TAA ATG/TAA ATG/TAA ATG/TAA ATG/TAA ATG/TAA ATG/TAA ATG/TAA ATG/TAA
ATG/TAA ATG/TAA ATG/TAA ATG/TAA ATG/TAA ATG/TAA ATG/TAA ATG/TAA ATG/TAA ATG/TAA ATG/TAA

ATG/TAA ATG/TAA ATG/TAA ATG/TAA ATG/TAA ATG/TAA ATG/TAA ATG/TAA ATG/TAA ATG/TAA ATG/TAA
ATG/TAA ATG/TAA ATG/TAA ATG/TAA ATG/TAA ATG/TAA ATG/TAA ATG/TAA ATG/TAA ATG/TAA ATG/TAA

ATG/TAA ATG/TAA ATG/TAA ATG/TAA ATG/TAA ATG/TAA ATG/TAA ATG/TAA ATG/TAA ATG/TAA ATG/TAA

Intergenic Nucleotide (bp)											Anti-Codon/One Letter
			P. cactorum				*pseudotsug*	*P. aleatoria*	*hedraiand*	*. clandestin*	
CH98PA11	ac_nanlinE	262HNP	CH9812411	CH98LOQ1	H02MKPy0	10300	P10339	NZFS 4037	CBS11172	P3942	
329	326	328	329	330	331	330	298	992	697	1466	
167	167	167	167	193	168	166	166	165	178	163	
56	60	56	56	60	60	60	60	55	55	55	
205	205	205	205	204	205	205	204	185	206	216	
37	38	38	38	38	38	38	38	38	38	35	
21	22	22	22	22	22	22	22	22	22	35	GTC
25	25	25	25	25	25	25	25	36	36	24	
59	59	59	59	59	59	59	59	57	57	55	
-26	-26	-26	-26	-26	-26	-26	-26	-26	-26	-26	
17	17	17	17	18	19	17	17	18	17	16	
2	2	2	2	2	2	2	2	2	2	3	TAC
0	1	1	1	1	1	1	1	1	1	1	GAT
8	10	10	10	10	10	10	10	10	10	11	TTG
3	4	4	4	4	4	4	4	4	4	4	GCG
18	18	18	18	18	18	18	18	18	18	18	
6	6	6	6	6	6	6	6	6	6	6	GAA
115	115	115	115	115	115	115	115	115	115	115	
10	11	10	10	10	11	10	10	9	11	12	
3	3	3	3	3	3	3	3	3	3	4	
24	25	25	25	25	25	24	25	27	25	26	GTG
30	30	30	30	30	30	30	30	30	30	24	
80	80	80	80	80	80	80	80	80	80	79	TTC
294	296	294	294	294	292	295	288	428	243	557	
43	43	43	43	43	43	43	43	43	43	41	
31	25	25	31	25	25	25	19	25	17	24	
694	649	648	649	649	649	648	649	820	667	684	TCT
2	2	2	2	2	2	2	2	2	2	3	
-4	-4	-4	-4	-4	-4	-4	-4	-4	-4	-4	
104	105	105	105	105	105	105	105	83	103	103	
9	10	10	10	9	10	10	10	11	10	10	TAG
23	23	23	23	23	23	23	23	22	23	22	TAA
218	190	218	218	190	190	213	190	7	244	207	

11	11	11	11	11	11	11	11	11	11	11	GCA
18	18	18	18	18	18	18	18	18	18	18	TGA
12	12	12	12	12	12	12	12	12	12	12	
28	28	28	28	28	28	28	28	28	28	28	
3	3	3	3	3	3	3	3	3	3	3	
3	3	3	3	3	3	3	26	3	3	3	
8	8	8	8	8	8	8	8	8	8	-23	
2	2	2	2	2	2	2	2	2	2	2	
15	15	15	15	15	15	15	15	15	15	22	CAT
61	61	61	61	61	61	61	61	61	61	61	
20	21	21	21	21	21	21	21	9	21	21	
2	3	3	3	3	3	3	3	3	3	3	TTT
17	18	18	18	18	18	18	18	18	18	18	TGC
10	10	10	10	10	10	10	10	8	8	8	
8	8	8	8	8	8	8	8	8	7	6	
6	6	13	6	6	6	6	6	6	6	6	
8	8	8	8	8	8	8	8	8	8	8	
9	9	9	9	9	9	9	9	9	9	2	
25	25	25	25	25	25	25	25	11	25	25	
5	6	6	6	6	6	4	6	6	6	6	
10	12	12	12	12	12	12	12	12	12	12	GTT
19	20	20	20	20	20	20	20	20	20	19	GCT
43	43	43	43	43	43	43	43	43	43	42	CAT
13	13	13	13	13	13	13	13	13	13	13	TGG
17	17	17	17	17	17	17	17	17	17	17	CAT
6	6	6	6	6	6	6	6	6	6	6	
6	7	7	7	7	7	7	7	7	7	7	
93	94	94	95	94	94	101	94	94	94	102	GCC
18	13	18	18	18	13	18	28	18	13	5	TCC
361	361	361	361	364	371	362	362	385	366	526	GTA
33	45	33	33	33	33	33	45	33	33	33	
423	492	423	423	496	496	493	353	459	413	1163	CCA
0	0	0	0	0	0	0	0	0	0	0	

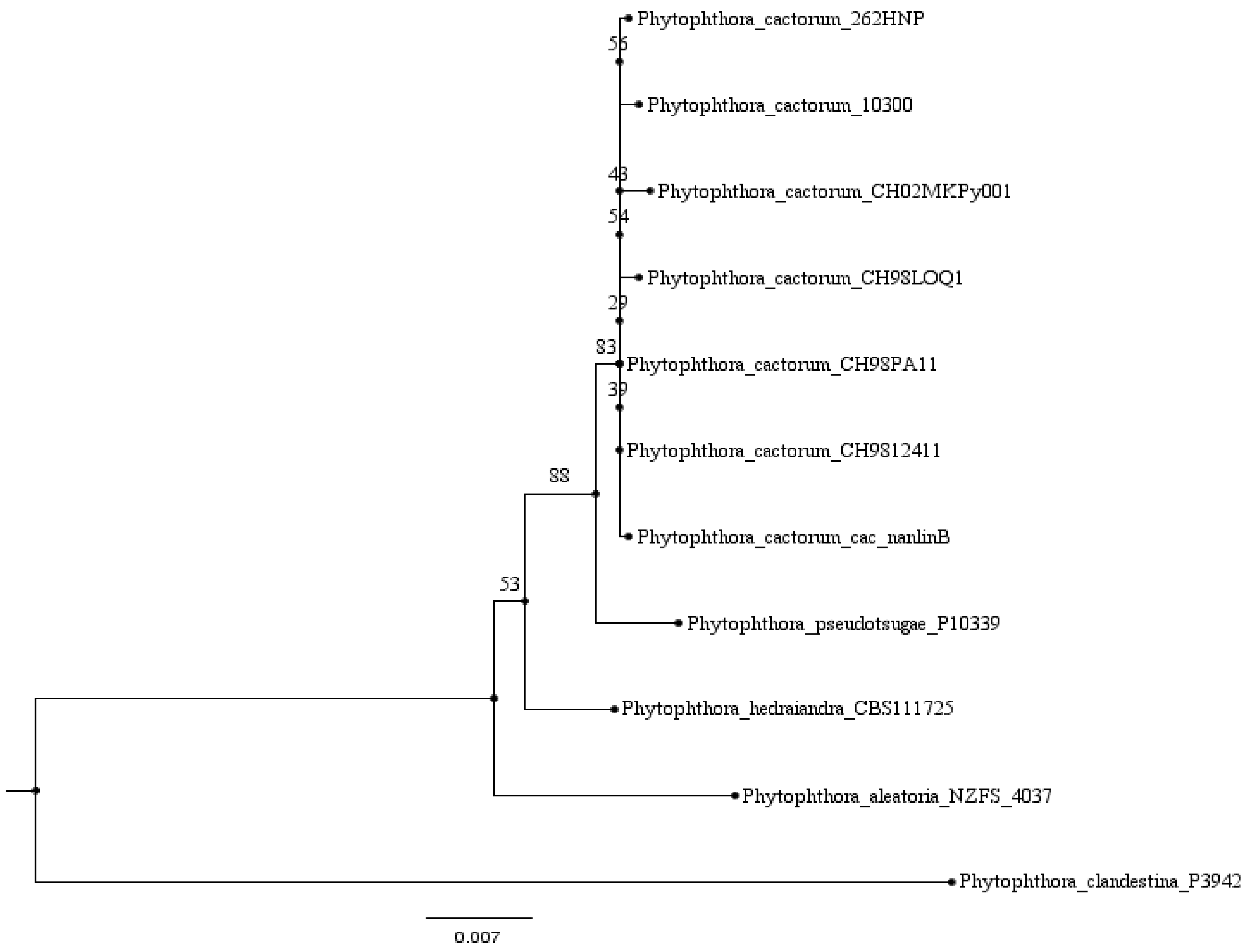

Figure S1 Phylogenetic tree of 11 *Phytophthora* strains constructed by Maximum Likelihood (ML) method based on the *cox1* gene sequences. *P. clandestina* was used as the outgroup. Numerals at nodes are ML bootstrap support values.

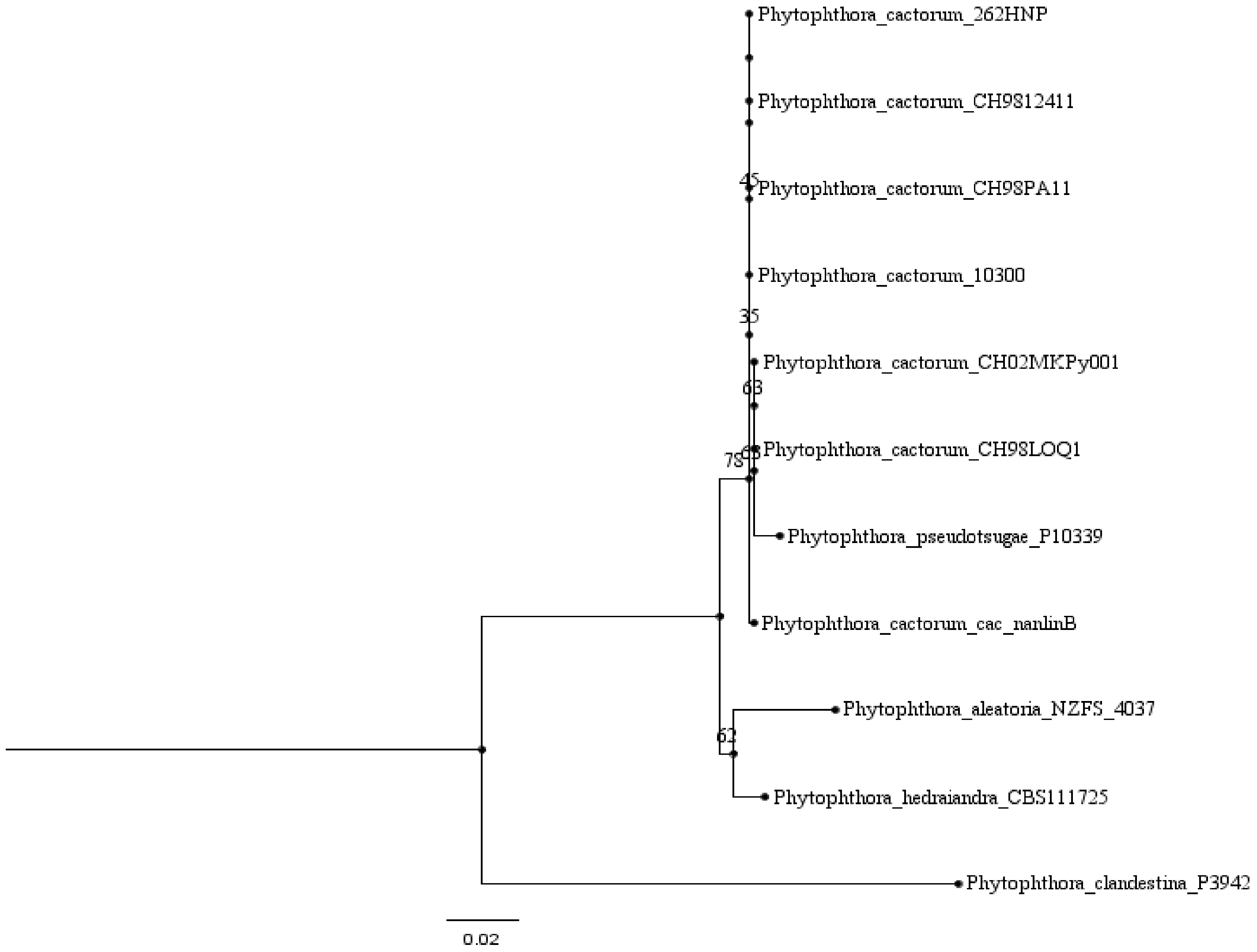

Figure S2 Phylogenetic tree of 11 *Phytophthora* strains constructed by Maximum Likelihood (ML) method based on the *cox2* gene sequences. *P. clandestina* was used as the outgroup. Numerals at nodes are ML bootstrap support values.

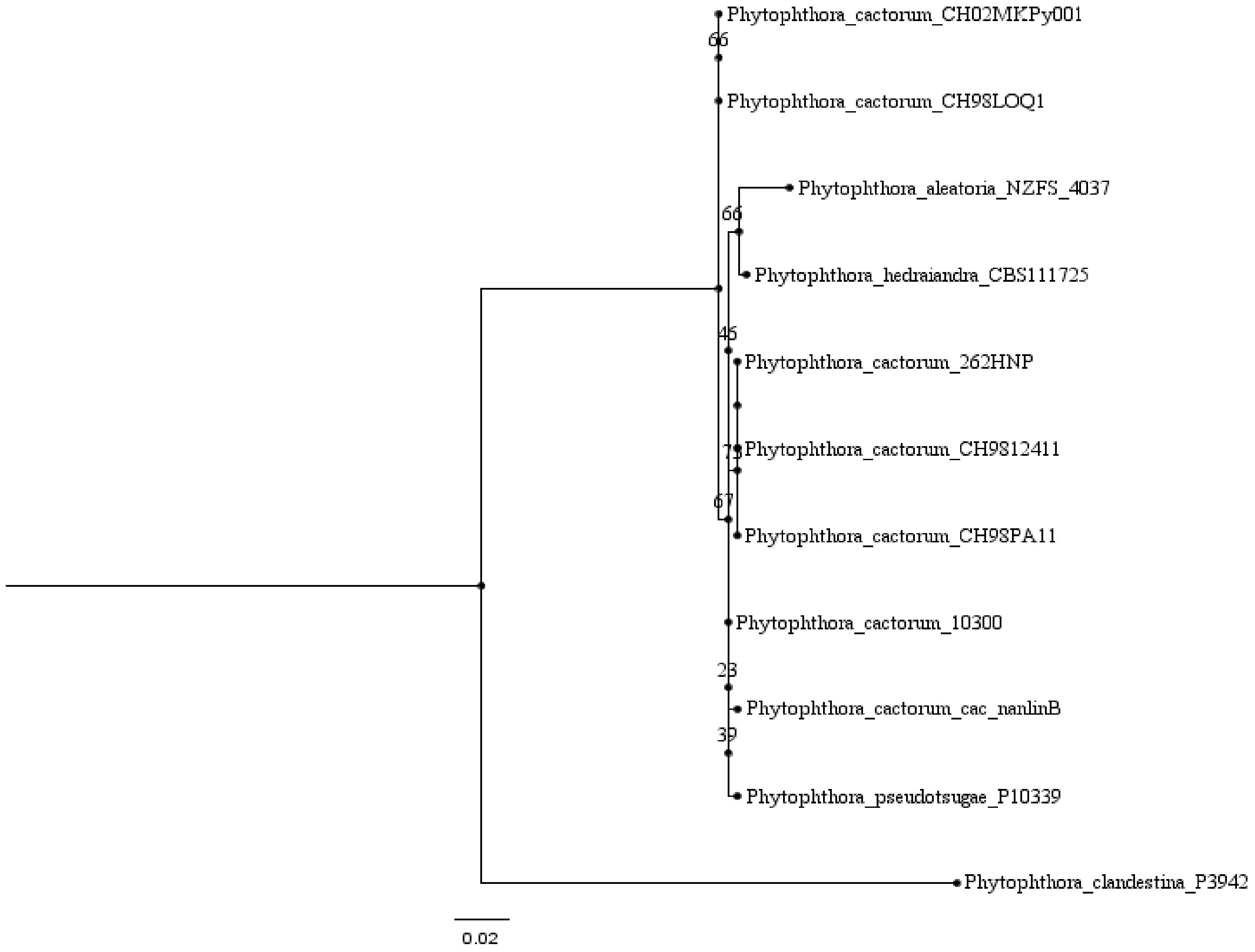

Figure S3 Phylogenetic tree of 11 *Phytophthora* strains constructed by Maximum Likelihood (ML) method based on the *rps10* gene sequences. *P. clandestina* was used as the outgroup. Numerals at nodes are ML bootstrap support values.

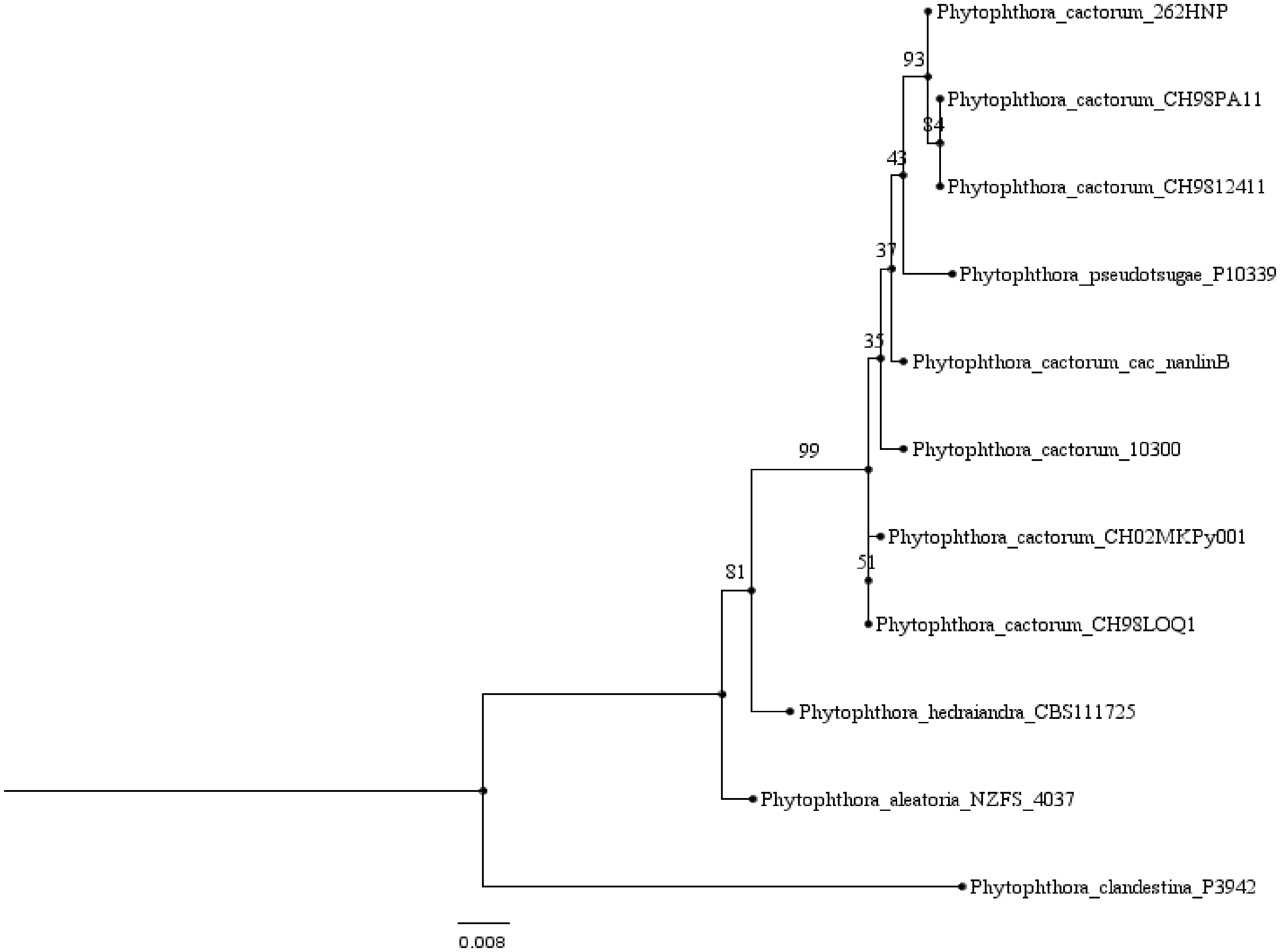

Figure S4 Phylogenetic tree of 11 *Phytophthora* strains constructed by Maximum Likelihood (ML) method based on the *nad9* gene sequences. *P. clandestina* was used as the outgroup. Numerals at nodes are ML bootstrap support values.

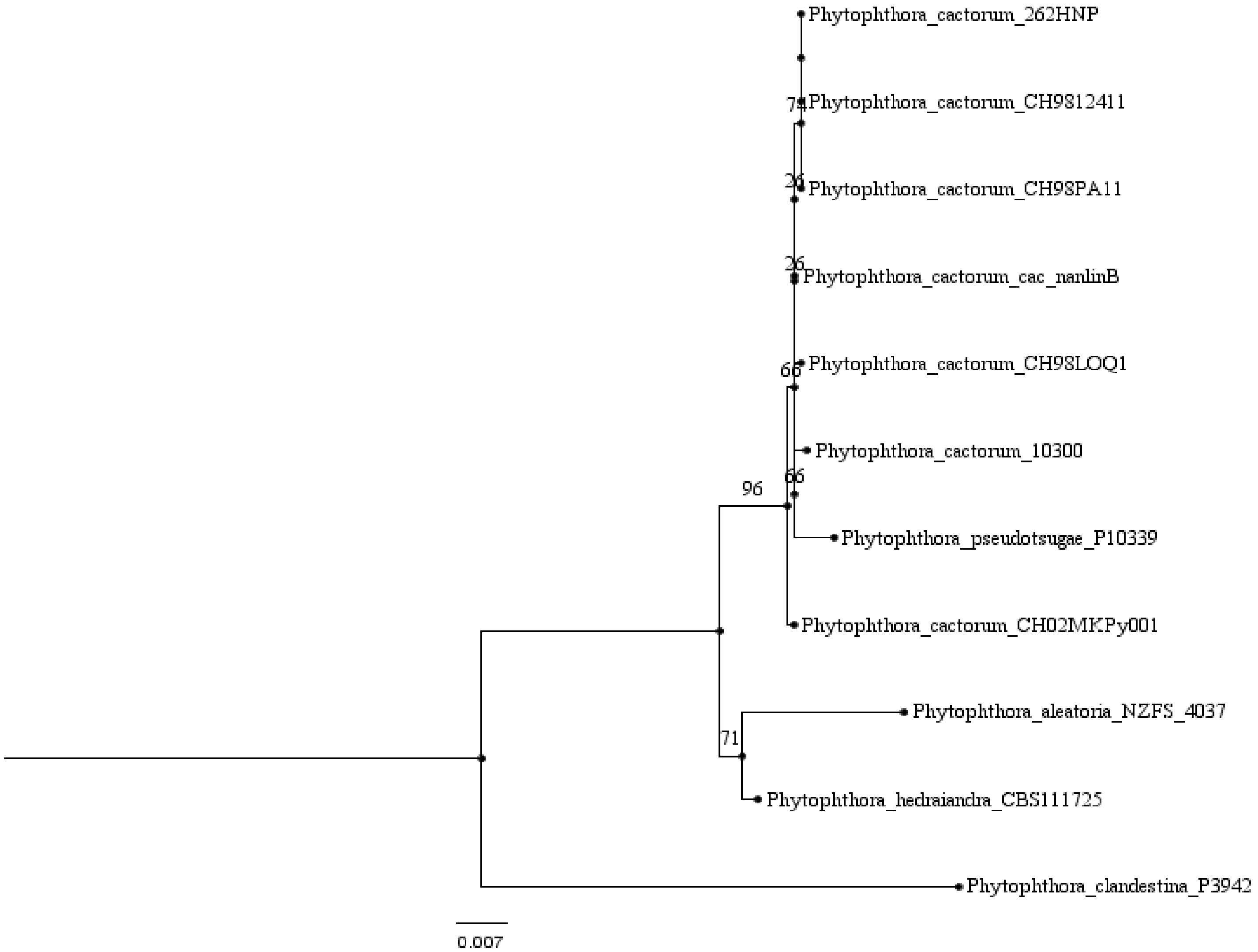

Figure S5 Phylogenetic tree of 11 *Phytophthora* strains constructed by Maximum Likelihood (ML) method based on the *cob* gene sequences. *P. clandestina* was used as the outgroup. Numerals at nodes are ML bootstrap support values.

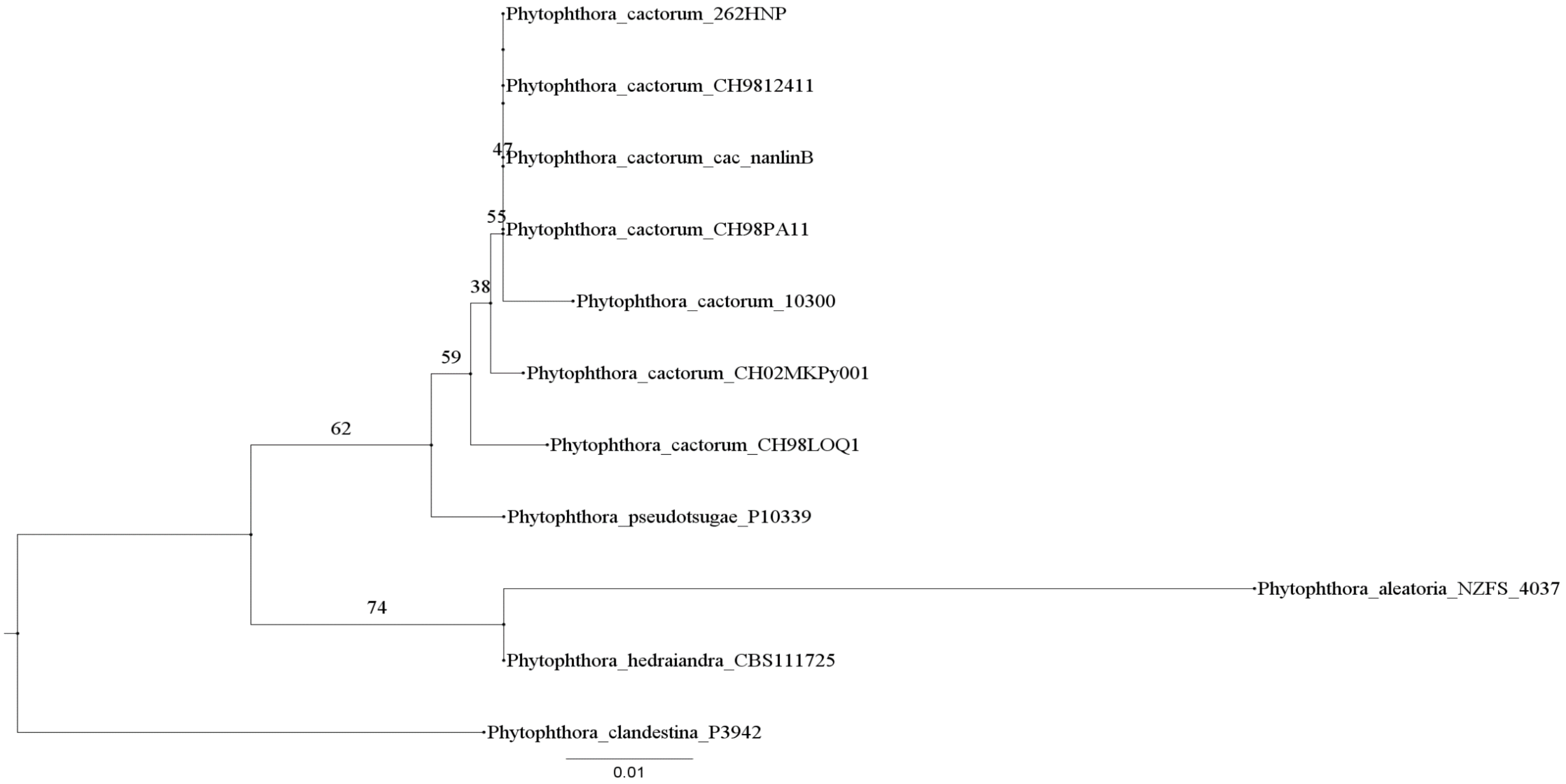

Figure S6 Phylogenetic tree of 11 *Phytophthora* strains constructed by Maximum Likelihood (ML) method based on the *rps12* gene sequences. *P. clandestina* was used as the outgroup. Numerals at nodes are ML bootstrap support values.

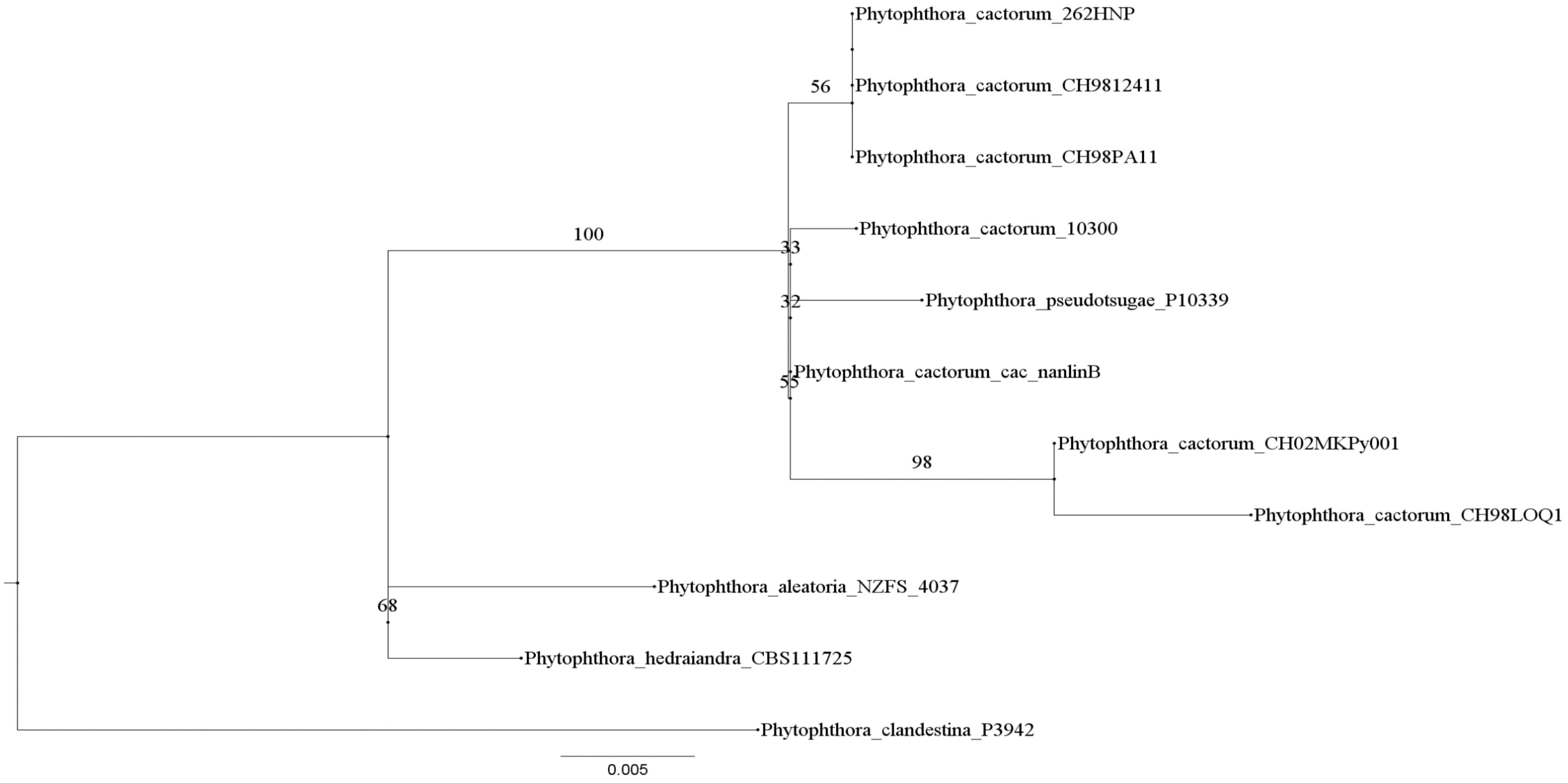

Figure S7 Phylogenetic tree of 11 *Phytophthora* strains constructed by Maximum Likelihood (ML) method based on the *rps13* gene sequences. *P. clandestina* was used as the outgroup. Numerals at nodes are ML bootstrap support values.

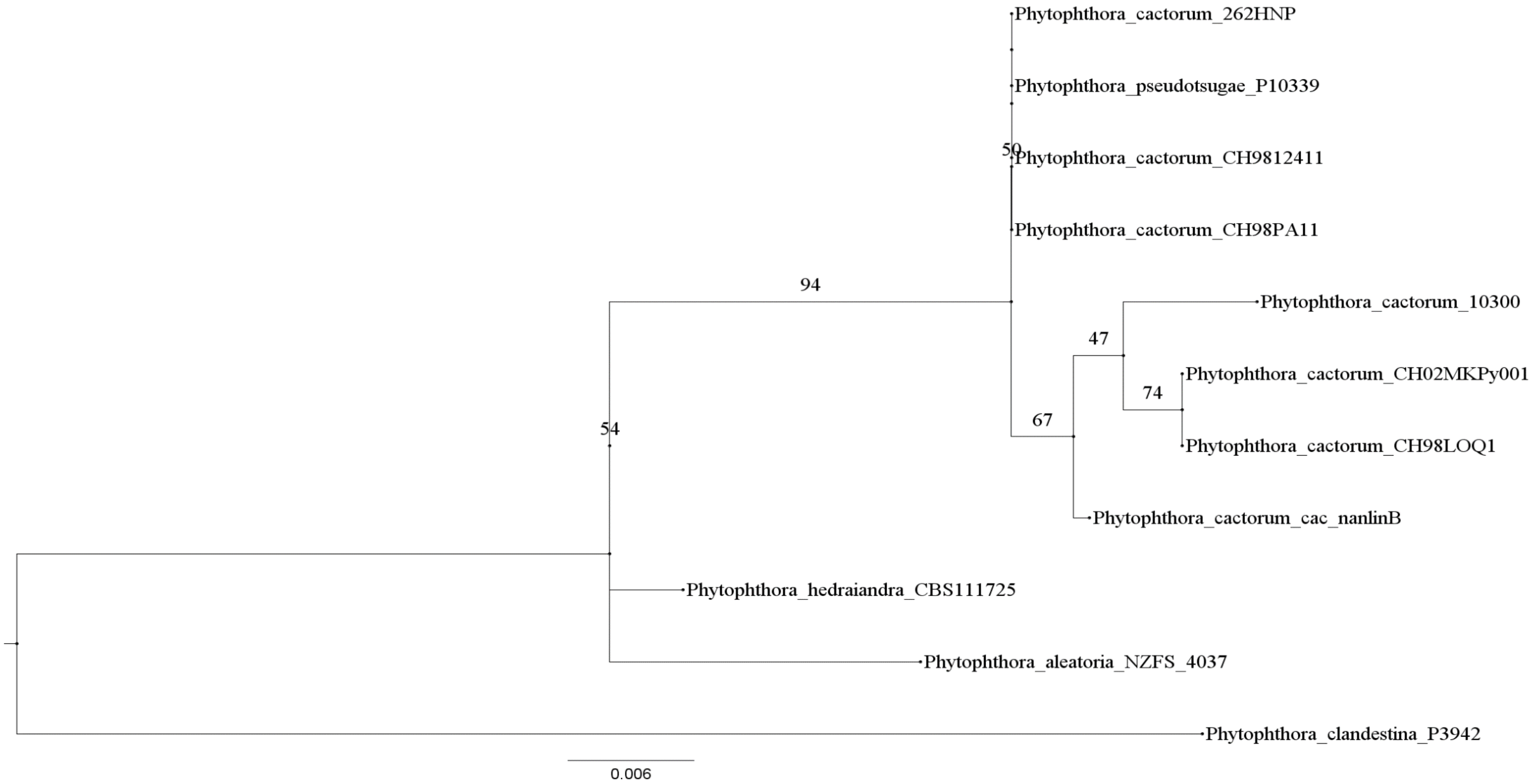

Figure S8 Phylogenetic tree of 11 *Phytophthora* strains constructed by Maximum Likelihood (ML) method based on the *rps14* gene sequences. *P. clandestina* was used as the outgroup. Numerals at nodes are ML bootstrap support values.
